# Intensive Care Unit Patient Outcome Prediction Using ν-Support Vector Classification and Stochastic Signal Processing–Based Feature Extraction Techniques: Algorithm Development and Validation Study

**DOI:** 10.2196/72671

**Published:** 2025-08-26

**Authors:** Shaodong Wang, Yiqun Jiang, Qing Li, Wenli Zhang

**Affiliations:** 1 Department of Industrial & Manufacturing Systems Engineering Iowa State University Ames, IA United States; 2 Robert D. and Patricia E. Kern Center for the Science of Health Care Delivery Mayo Clinic Rochester, MN United States; 3 Department of Information Systems and Business Analytics Iowa State University Ames United States

**Keywords:** health care operation management, stochastic signal analysis, machine learning, intensive care unit outcome prediction, health digital traces, feature engineering

## Abstract

**Background:**

Intensive care units (ICUs) treat patients with life-threatening illnesses. Worldwide, intensive care demand is massive. Predicting patient outcomes in ICUs holds significant importance for health care operation management. Nevertheless, it remains a challenging problem that researchers and health care practitioners have yet to overcome. While the newly emerging health digital trace data offer new possibilities, such data contain complex time series and patterns. Although researchers have devised severity score systems, traditional machine learning models with feature engineering, and deep learning models that use raw clinical data to predict ICU outcomes, existing methods have limitations.

**Objective:**

This study aimed to develop a novel feature extraction and machine learning framework to repurpose and extract features with strong predictive power from patients’ health digital traces for ICU outcome prediction.

**Methods:**

Guided by signal processing techniques and medical domain knowledge, the proposed framework introduces a novel, signal processing–based feature engineering method to extract highly predictive features from ICU digital trace data. We rigorously evaluated this method on a real-world ICU dataset, demonstrating significant improvements over both traditional and deep learning baseline methods. The method was then evaluated using a real-world database to assess prediction accuracy and feature representativeness.

**Results:**

The prediction results obtained by the proposed framework significantly outperformed state-of-the-art benchmarks. This demonstrated the framework’s effectiveness in capturing key patterns from complex health digital traces for improving ICU outcome prediction.

**Conclusions:**

Our study contributes to health care operation management by leveraging digital traces from health care information systems to address challenges with significant implications for health care.

## Introduction

### Background

As the Fourth Industrial Revolution unfolds, health care organizations worldwide are implementing an increasing number of digital artifacts capable of producing or collecting data to modernize services, scale business, and improve the efficiency of information exchange. These digital artifacts generate and record vast quantities of data regarding the conditions and outcomes of patients, enabling the digital tracing of these individuals [1]. These digital traces offer a rich collection of novel and valuable sociotechnical empirical data. This abundance of new information can greatly enhance decision-making processes in health care applications. As part of this tendency, the implementation of electronic intensive care unit (eICU) technology over the last decade has allowed large amounts of intensive care unit (ICU) patients’ vital sign data (ie, a group of medical signs that indicate the status of the body’s life-sustaining functions, such as blood pressure, heart rate, and respiratory rate) to be collected and streamed [2]. These real-time time-series data, originally used to monitor patients’ real-time conditions, coupled with other patient information recorded in health IT systems (eg, demographics), constitute ICU patients’ health digital traces.

ICUs are hospital departments dedicated to providing critical care medicine to patients who are at risk of, currently experiencing, or recovering from life-threatening illnesses or injuries. ICU patients are extremely vulnerable to adverse outcomes due to their rapid disease progression and have the highest mortality rate of all patients across different health care departments [3]. Worldwide, intensive care demand is massive. Researchers and health care practitioners have long recognized the significance of ICU outcome prediction, which is generally defined as predicting patient outcomes resulting from medical treatment in the ICU, including but not limited to patient mortality, length of stay, readmission, morbidity, disability, and quality of life [4]. It has significant implications on health care operation management, such as laying the scientific foundation for assessing the severity of illness, providing a standard for adjudicating new treatments and policies, providing a way for comparing cohorts of ICU patients treated across different hospitals and countries, allocating resources and determining levels of care, and discussing expected outcomes with ICU patients and families [5,6].

However, predicting ICU outcomes is a complex problem that practitioners and researchers have yet to overcome. ICU patients have diverse and dynamic characteristics; they come from various diagnosis cohorts, have unique demographics and disease progressions, and may receive different levels of medical interventions [7,8]. Effectively identifying patterns and predicting patients’ ICU outcomes poses great challenges in health care analytics. The emergence of eICUs during the Fourth Industrial Revolution, along with the availability of patients’ digital health data from eICUs, has created new opportunities for developing more sophisticated methods for predicting ICU outcomes. Researchers have demonstrated that, in addition to being used for monitoring purposes, ICU patients’ health digital traces contain rich dynamic patterns that can be repurposed to inform prognosis, provide early forecasts of life-threatening conditions, and predict patient outcomes [9]. Many researchers who work on ICU outcome predictions have explored the value of patients’ health digital traces by incorporating real-time vital sign data as the input of traditional machine learning models with feature engineering or deep learning models using raw clinical data. However, both types of methods have limitations. This is because ICU patients’ health digital traces include complex time-series data and patterns [8]. Current feature engineering–based traditional machine learning models rely largely on simple summary statistics of vital signs and are incapable of capturing heterogeneous and dynamic patterns from patients’ health digital traces, resulting in unsatisfying performance. On the other hand, deep learning models rely heavily on computational power and large amounts of training data, which are normally not available for health care predictive tasks [10]. This is because the integration of patient data for a prediction task in health care analytics must be executed with great care (eg, considering different patient cohorts and different periods), making it impractical to acquire a sufficient amount of training data for complex deep learning models. Researchers and practitioners urge the next generation of ICU outcome prediction models to be more accurate (predict with better performance), autonomous (execute without time-consuming or manual data entry), and dynamic (capture temporal changes in physiological signals and clinical events) [4,11]. Using mortality prediction as a research case, the objective of this study was to develop a new method that aims to extract meaningful patterns from readily available health digital traces to facilitate accurate ICU outcome predictions.

To achieve this goal, we repurposed and used ICU patients’ health digital trace data from the eICU systems as input. To effectively extract patterns from the complex time series of vital signs in patients’ health digital traces, we then used signal processing techniques to decompose the time-series data, enhance useful signals, and reduce noise in complex time series. Next, guided by medical domain knowledge and feature selection techniques, we identified the most representative features from the decomposed health digital trace data for ICU mortality prediction. Finally, using a state-of-the-art machine learning technique, the proposed framework accurately predicted the mortality rate for ICU patients. To demonstrate the effectiveness of the proposed framework, we evaluated it on a large real-world ICU database. The proposed method outperformed strong baseline methods, including the Acute Physiology and Chronic Health Evaluation (APACHE) IV model (ie, the best-performing scoring system in ICU outcome prediction that is already used in hospitals), time-series forecasting methods (ie, autoregressive moving average [ARMA] and autoregressive integrated moving average [ARIMA]), other traditional machine learning models with statistical features, and deep learning models (ie, convolutional neural networks [CNNs], long short-term memory [LSTM], and gated recurrent unit [GRU]), by a large margin.

Our main contributions are as follows: (1) we propose a new feature engineering framework that leverages stochastic signal processing and medical domain knowledge to extract predictive features from ICU digital traces; (2) we designed a structured feature selection process to enhance model interpretability and prediction accuracy; (3) through extensive experiments, we demonstrated that our method significantly outperforms traditional statistical and deep learning models on ICU mortality prediction tasks; and (4) we showed that the features extracted by our framework generalize across patient cohorts and can be integrated into existing clinical decision systems. Moreover, our work has practical implications for ICU outcome prediction and health care operation management: (1) it requires only readily available digital health trace data from ICU bedside monitors rather than laboratory results and intensivists’ assessments, (2) it significantly improves the performance of ICU mortality predictions, and (3) the extracted features can effectively represent heterogeneous ICU patient cohorts.

### Related Work

#### ICU Outcome Prediction and Limitations of Extant Studies

The existing methods for predicting ICU outcomes can be classified into 3 main types (Table 1): severity scoring systems, traditional machine learning models with feature engineering, and deep learning models with raw clinical data. For severity scoring systems, the most reputable ones (including major revisions of these models) are the APACHE [12], Simplified Acute Physiology Score [13], and Mortality Probability Model [14]. Among the existing severity scoring systems, APACHE IV demonstrates the highest performance in terms of area under the curve (AUC) [15]. Despite their widespread use, the reliability of the severity scoring systems, including APACHE IV, has been questioned by practitioners [4]. More importantly, there are ongoing concerns about the prolonged waiting time of laboratory data collection and the assessments needed from subject matter experts for calculating the severity scores [11]. For instance, APACHE IV requires 24 hours to gather all the necessary information for prediction. The predicting variables include laboratory test results and Glasgow Coma Scale (GCS) measures—the laboratory test results can take hours to days to obtain depending on the complexity of the tests [16], the GCS scores necessitate expert medical evaluation, and their reproducibility has raised concerns among researchers [11]. Researchers argue that the next generation of ICU mortality predictive models should use an automated electronic system for data gathering and prediction generating [4,11].

**Table 1:** Summary of intensive care unit (ICU) mortality prediction models from the literature.

Category and representative method		Required resources				Research gaps	
		Limited resources		Readily available health digital traces			
		Laboratory test results	Intensivist assessment^a^	Pre-ICU conditions	Vital signs		
**Severity scoring system^b^**							Low accuracy; requires expert assessments and laboratory test results; unable to conduct real-time forecasting
	SAPS^c^ III	Yes	Yes	No	Statistics features		
	APACHE^d^ IV	Yes	Yes	Yes	Statistics features		
	MPM^e^ III	No	Yes	Yes	Statistics features		
**Traditional machine learning model with feature engineering^f^**							Lack of effective means to extract meaningful patterns from complex time series
	DT^g^, SVM^h^, NN^i^, and LR^j^	Yes	Yes	No	Statistics features		
	D-TSK-FC^k^	Yes	No	No	Statistics features		
	RF^l^, LR, NN, and SVM	Yes	Yes	No	Statistics features		
	RF, GB^m^, and LR	Yes	Yes	No	Statistics features		
	SVM, GB, XGBoost^n^, and LR	Yes	Yes	No	Statistics features		
**Deep learning model with raw clinical data^o^**							Relies on computational power and large amounts of training data
	CNN^p^ model 1	Yes	Yes	No	Time series		
	CNN model 2	No	No	No	Time series		
	LSTM^q^	No	Yes	Yes	Time series		

^a^Glasgow Coma Scale.

^b^Zimmerman et al [12], Moreno et al [13], and Higgins et al [14].

^c^SAPS: Simplified Acute Physiology Score.

^d^APACHE: Acute Physiology and Chronic Health Evaluation.

^e^MPM: Mortality Probability Model.

^f^Davoodi and Moradi [17], Kim et al [18], Hsieh et al [19], Kong et al [20], and Zhai et al [21].

^g^DT: decision tree.

^h^SVM: support vector machine.

^i^NN: neural network.

^j^LR: logistic regression.

^k^D-TSK-FC: deep Takagi-Sugeno-Kang fuzzy classifier.

^l^RF: random forest.

^m^GB: gradient boosting.

^n^XGBoost: extreme gradient boosting.

^o^Caicedo-Torres and Gutierrez [22], Kim et al [23], and Thorsen-Meyer et al [24].

^p^CNN: convolutional neural network.

^q^LSTM: long short-term memory.

With the emergence of health digital trace data, researchers have recognized the potential of such data in enhancing ICU outcome prediction [9]. This is because these data reveal patients’ pathological conditions and their response to treatments, making them valuable for improving prediction performance. Traditional machine learning models have been adopted for ICU outcome predictions using patients’ digital trace data, which have included demographic information and summary statistics of vital measurements (Table 1). Despite researchers continuously introducing various prediction models, the features extracted from the health digital traces remain relatively simple—basic statistics of vital sign time series, such as the minimum and maximum respiration rates or blood pressure. However, there is increasing evidence suggesting that superior accuracy in ICU outcome prediction requires more effective feature extraction methods [4]. The complexity of patient cohorts’ heterogeneity and the complexity of the time series of health digital traces pose significant challenges in extracting meaningful dynamic patterns and uncovering the relationships among these patterns.

Deep learning models with strong pattern recognition capabilities are also used in ICU outcome prediction. CNNs, which can summarize patterns from patients’ health digital traces, have been implemented first [22,23]. Researchers also input patients’ vital sign data into recurrent neural networks (RNNs) to infer ICU outcomes, which takes advantage of the temporal information of vital signs [24]. However, these models take the entire time series of vital signs as input, and their performance greatly depends on computer power and massive amounts of training data, which is challenging in health care practice [10]. In health care predictive analyses, the integration of patient data must be executed with great care, making it impractical to acquire sufficient training data for complex deep learning models. The integration of health care data from different patient cohorts (eg, various diseases, distinct ICU admission types, different races, and diverse age groups) must be undertaken with meticulous care. For example, patients with different genetic backgrounds (ethnicities) are sometimes susceptible to certain diseases; patient cohorts comprising geriatric, neonatal, and general patients show notable variations in disease risks and prognosis. These differences significantly influence health care prediction results. In ICU outcome prediction, it is often necessary to separate the different patient cohorts instead of integrating their data. There is also an inherent temporal aspect to patient data, and it is not appropriate to integrate patient data from vastly different periods. Societal development changes patients’ physical fitness, underlying health conditions, and health care providers’ treatments, leading to significant variations in patient data distribution. Overall, acquiring sufficient training data for complex deep learning models is usually impractical for health care predictive analytics. Consequently, the performance of complex deep learning models is constrained by the limitations of available training data (experiments are provided in Postanalysis: The Impact of Limited Patient Data on Deep Learning Model Performance section).

As ICU patients’ health digital traces contain complex time-series data, statistical forecasting models such as ARMA and ARIMA may also be used to analyze the time-series data. However, these time-series models are developed to predict the value of the time series at the next time step and are not created for prediction or classification tasks or probability estimations. To make predictions using time-series data, researchers regard the coefficients of the time-series models as input features and train machine learning classifiers [25]. Nevertheless, these methods are not ideal for the time series of patients’ health digital traces from ICUs. The order of a time-series model has to be determined by the statistical characteristics of a specific time series (eg, one time series of vital signs from a specific patient). Researchers usually treat model orders as hyperparameters and determine them through experiments and the Akaike information criterion; a fixed order of time-series models is required for all patients to ensure that the input features have the same dimension for the classification task, which limits the predictive power of the time-series forecasting models in ICU outcome prediction.

#### ICU Patients’ Health Digital Traces and Stochastic Signal Analysis Techniques

The health digital traces of ICU patients have been originally used for monitoring and assessing patients’ immediate well-being. A growing body of literature has shown that many shared dynamic patterns can be identified across heterogeneous patient cohorts that may be repurposed to evaluate illness severity, identify future clinical abnormalities, predict adverse events, or distinguish heterogeneous patient cohorts [9]. However, identifying and extracting meaningful features from health digital traces remains a challenging task given that the range of a digital trace varies with a patient’s age, gender, weight, environment, medical condition and intervention, and many other factors [9]. As a result, the health digital traces contain complex time series and exhibit diverse and dynamic patterns. As we later demonstrate (refer to the Results section), extant feature extraction and ICU outcome prediction methods are inadequate.

Stochastic signal processing, a field of science concerned with processing and analyzing time-series data, is a well-suited tool to extract complicated patterns of time-series digital traces. Stochastic signal processing techniques are particularly useful for extracting patterns from time-series signals, which are normally described as aperiodic, noisy, intermittent, and transient [26]. They differ from other time-series analysis tools for 2 reasons. First, they examine the signal in both the time domain (ie, the time series of patients’ health digital traces) and the frequency domain (ie, the magnitude of change within each frequency band of the time series) simultaneously. Therefore, they have powerful capabilities for enhancing the useful signals in complex time series and increasing the signal-to-noise ratio, which facilitates feature extraction from patients’ digital traces. Second, they have computational algorithms that reduce the computing time and complexity of large transformations, so the time-series data can be processed almost instantaneously.

Although complex time series in patients’ health digital traces can be decomposed using signal processing techniques for noise reduction and signal enhancement, specific domain knowledge is required to determine how to extract meaningful patterns from the decomposed representations of health digital traces. In health care research, medical diagnosis signals, including signals from electrocardiograms (ECGs), electroencephalograms, and photoplethysmogram, are analyzed using signal processing techniques based on researchers’ and practitioners’ medical knowledge in beat-to-beat heart rate patterns, electrical activity in the brain, and optical signals in blood volume changes [26]. These studies show the potential of adapting stochastic signal processing techniques in health care analytics research. However, in these existing studies, signal processing has been used for specific diagnostic purposes, with an emphasis on explanation rather than prediction. In this study, we sought to combine medical knowledge regarding the patterns and variability of ICU patients’ vital signs to extract meaningful features for predicting ICU outcomes. To our knowledge, the complicated time series of vital signs in patients’ digital traces have never been systematically analyzed using signal processing techniques. Combining medical domain knowledge, the proposed method provides a novel strategy to extract predictive features for improved ICU outcome prediction results.

To summarize, the deficiencies of existing ICU mortality prediction methods, coupled with the challenges associated with leveraging patients’ health digital traces contained in complex time series, motivate us to propose a new method that can be used to (1) effectively extract representative features from ICU patients’ digital trace data and (2) accurately predict ICU mortality using readily available data.

#### Feature Engineering in ICU Outcome Prediction

In previous ICU prediction literature, feature engineering has predominantly focused on extracting basic statistical descriptors from vital signs, such as minimum, maximum, mean, and SD [18-21]. These summary statistics provide coarse information about the central tendency and spread of physiological signals but often overlook dynamic temporal and spectral patterns.

Meanwhile, signal processing techniques such as wavelet transforms (WTs), spectral analysis, and autocorrelation have been explored in predictive modeling for specific signals (eg, most notably from ECGs) for tasks such as arrhythmia classification, early warning score prediction, and ICU mortality estimation [26-29]. However, these studies generally target a narrow range of signals and transformations. Our work expanded on this by applying a broader set of signal decomposition methods (fast Fourier transform [FFT], power spectral density [PSD], autocorrelation, and WT) across multiple ICU vital signs (eg, arterial oxygen saturation [SaO_2_], heart rate, and respiration) and by combining the results with clinical insights to guide feature design. Moreover, we introduced new composite features, such as power in band and relative extrema, that more precisely quantify signal variability and instability, both of which are clinically meaningful. This approach results in a diverse and interpretable feature set that enhances the model’s ability to predict ICU outcomes across heterogeneous patient cohorts.

To ensure that these engineered features are clinically relevant, we grounded our signal processing techniques in established medical knowledge. First, for heart rate variability, we computed power within the low-frequency and high-frequency bands using PSD analysis. These frequency bands are associated with sympathetic and parasympathetic nervous system activities, respectively, and are critical in assessing autonomic function in patients who are critically ill [30]. Second, given the nonstationary nature of physiological signals such as ECG and respiratory patterns, we used WT to capture transient features and localized frequency components. This approach facilitates the detection of clinically significant events such as arrhythmias and respiratory irregularities. Notably, unstable respiration can lead to respiratory muscle fatigue, cardiovascular collapse, and impaired oxygen delivery [31]. Third, features such as relative extrema were designed to identify sudden changes in vital signs, such as abrupt drops in peripheral oxygen saturation or spikes in heart rate, which may indicate acute clinical events. Similarly, power-in-band features help in quantifying the energy within specific frequency bands associated with pathological conditions [32]. Fourth, by aligning our signal processing techniques with established medical knowledge, we aimed to extract features that are not only statistically robust but also clinically interpretable, thereby enhancing the utility of our predictive models in real-world ICU settings.

## Methods

**Figure 1:**
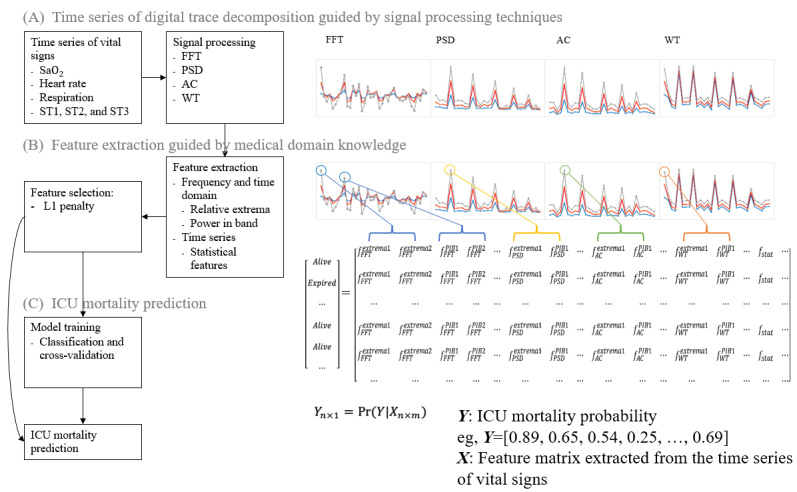
The proposed intensive care unit (ICU) mortality prediction framework. AC: autocorrelation; FFT: fast Fourier transform; PSD: power spectral density; SaO2: arterial oxygen saturation; ST1: estimated ST segment level 1 of the electrocardiogram (ECG); ST2: estimated ST segment level 2 of the ECG; ST3: estimated ST segment level 3 of the ECG; WT: wavelet transform.

We propose a novel method to effectively extract features with strong predictive power from the complex time series of health digital traces for ICU mortality prediction. As shown in Figure 1, the proposed model includes three steps: (1) time series of digital trace decomposition guided by signal processing techniques, (2) feature extraction guided by medical domain knowledge, and (3) ICU mortality prediction using ν–support vector classification (SVC).

### Time Series of Health Digital Trace Decomposition Guided by Signal Processing Techniques

#### Overview

ICU patients’ health digital traces contain multiple complex time series; each time series is denoted by *v_t_* (*t* is the time index and *t*≤*N*). To enhance useful signals and reduce noise in *v_t_*, in the first step, guided by signal processing techniques (Table 2), we decomposed *v_t_* using FFT, PSD, autocorrelation, and WT.

**Table 2:** Signal processing techniques and relations to health digital traces.

Technique	Signal processing guidelines^a^	Motivation and relation to health digital traces
FFT^b^	Using FFT, any time series can be decomposed into a series of simple sinusoids of different frequencies. The FFT estimates the coefficients of each sinusoid for a given time series.	To decompose complex health digital traces into several relatively milder, more regular, and stable subsequences
PSD^c^	The PSD describes the distribution of the power of a time series over frequency. FFT is great at analyzing vibration when there are a finite number of dominant frequency components, but PSDs can be used to characterize random vibration signals.	To analyze the random vibration signals, which are common in patients’ health digital traces.
AC^d^	AC is the correlation of a time series with the lagged version of itself over successive time intervals, which is usually used to detect repeating patterns, such as periodic signals hidden in noisy data.	To detect and enhance repeating patterns in patients’ health digital traces and reduce noise.
WT^e^	The WT decomposes a time series into a series of wavelets with different scales at different time points. Thus, the outputs of WT present both the strength and location of frequencies (ie, patterns from both the frequency and time domains) in the time series.	To include the information of the frequencies’ time location (time domain) as the outputs of the aforementioned 3 techniques (FFT, PSD, and AC) mainly provide information about the frequencies (frequency domain) in time-series data. Time domain information reveals patients’ disease or condition progression.

^a^Addison [26], Bloomfield [27], Woyczynski [28], and Broersen [29].

^b^FFT: fast Fourier transform.

^c^PSD: power spectral density.

^d^AC: autocorrelation.

^e^WT: wavelet transform.

The decomposed *v_t_* is denoted as *F*(ω) (ie, frequency spectrum), where ω is the parameter of the signal processing. Specifically, ω indicates frequency in FFT and PSD, scale and shift parameters in WT, and time difference in autocorrelation. For FFT, PSD, and autocorrelation, the frequency spectrum of a time series *v_t_* is a vector, [*F*(ω_1_), *F*(ω_2_),..., *F*(ω*_t_*)]; for WT, the frequency spectrum is a matrix ([*F*(ω_1,1_), *F*(ω_1,2_),..., *F*(ω_1,_*_t_*)], [*F*(ω_2,1_), *F*(ω_2,2_),..., *F*(ω_2,_*_t_*)],..., [*F*(ω*_s_*_,1_), *F*(ω*_s_*_,2_),..., *F*(ω*_s_*_,_*_t_*)]) where *s* is the number of rows decided by the scale of the WT. All frequency spectrums converted from time series of patients’ health digital traces form a space *X_n_*_×_ *_w_* (*n*=number of patients; *w*=the number of frequency spectrums). The following paragraphs introduce the signal processing transfer processes in our research setting.

#### FFT Process

The Fourier transformation of a signal reveals periodicity in time-series data and indicates the frequencies of these periodical components. The resulting signals after the FFT are frequency spectrums 
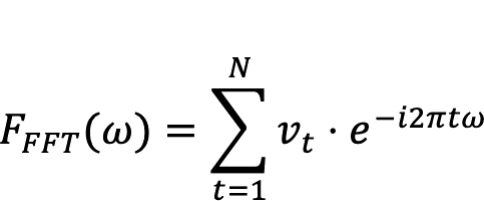
, where *v_t_* is the vital sign and ω is the frequency at which a complex sinusoid is computed.

#### PSD Process

The PSD *F_PSD_*(ω) is calculated using 
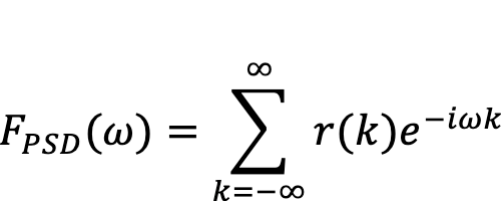
 where *r*(*k*)is the autocovariance sequence of *v_t_* and 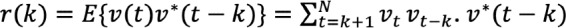 denotes the complex-conjugate transpose of *v*(*t* – *k*). The PSD characterizes the average power (ie, measure of signal strength) at a frequency ω in the signal. Specifically, for time-series data, the PSD uses the signal’s autocorrelations to measure the power. Compared to FFT, which obtains the amplitudes of a signal’s frequency components, the PSD of the signal delineates the power contained within the signal as a function of frequency.

#### Autocorrelation Process

Autocorrelation measures the correlation between a signal and its delayed version with lag ω, which can be calculated using 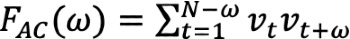. It reveals the influence of the previous signal on the following signal in the sequence. When the signal does not repeat the sequence of values regularly after a fixed length of time, the autocorrelation coefficients tend to be small, which indicates the fluctuation of *v_t_*. Otherwise, the autocorrelation coefficients tend to be large, which represents the stable status of health digital traces.

#### WT Process

The WT analyzes signals with a dynamic frequency spectrum, providing a high resolution in both the frequency domain and the time domain. The WT of the vital sign signal *v_t_* is expressed using 
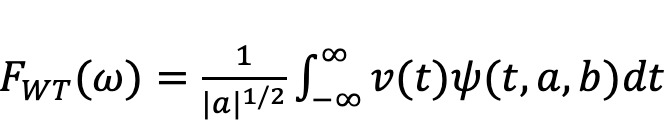
, where ω = (*a*, *b*) and ψ(·) is the mother wavelet (ie, a wavelike oscillation). Parameter *a* defines the scale (ie, how stretched a wavelet is) of the wavelet, and parameter *b* defines the time location (ie, where the wavelet is positioned in time) of the wavelet. We used 3 types of wavelets to generate frequency spectrums: Morlet wavelets (
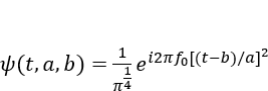
), complex Morlet wavelets (
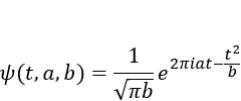
), and Mexican wavelets (
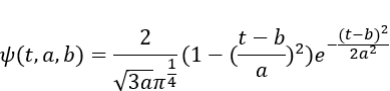
). Morlet and complex Morlet wavelets were included because they are closely related to human perception of vision. Mexican wavelets were used as they are widely used as broad-spectrum source terms in WT analysis.

### Feature Extraction Guided by Medical Domain Knowledge

#### Overview

Although signal processing techniques can enhance useful signals from ICU patients’ health digital traces that contain aperiodic, noisy, intermittent, and transient time series, the results from signal processing, *X_n_*_×_ *_w_*, are not ideal to use as input features of machine learning classifiers for ICU mortality prediction due to their high dimensionality. For predicting ICU outcomes, the valuable patterns are still hidden in the vast amount of information. Therefore, we extracted the most representative features from *X_n_*_×_ *_w_* for ICU outcome prediction by combining medical knowledge regarding the patterns and variability of ICU patients’ vital signs (Multimedia Appendix 1). In addition, we took various statistical features from the time series *v_t_*. The extracted features formed a new feature space, *X_n_*_×_ *_l_* (*n*=number of patients; *l*=the number of features), where *l*<<*w* We evaluated the relative importance of the extracted features and selected those with the highest predictive power for ICU mortality. The selected features, *X_n_*_×_ *_m_* (*n*=number of patients; *m*=the number of selected features), were the input of the proposed ICU mortality prediction model.

#### Relative Extrema

**Figure 2:**
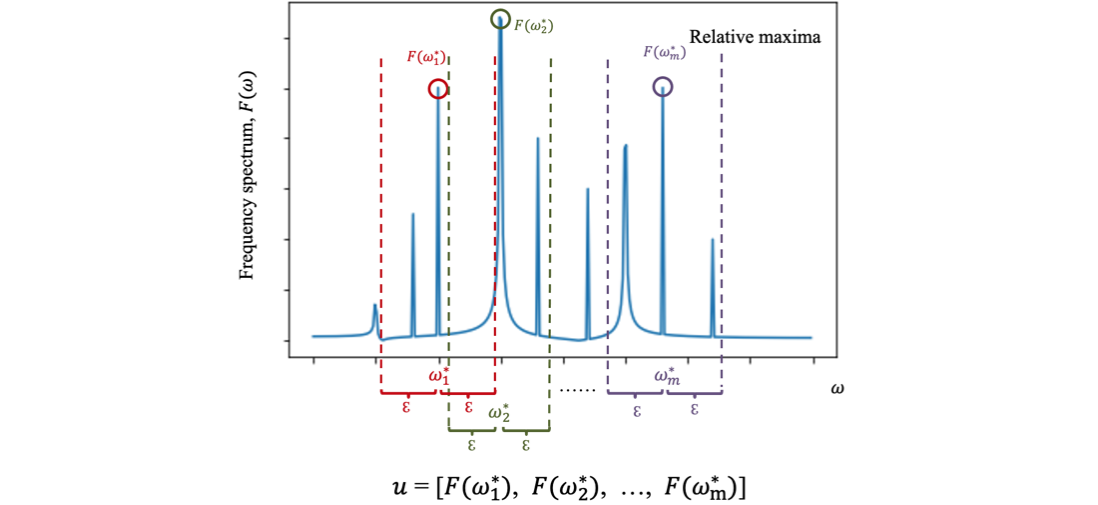
An example of relative maxima of the frequency spectrum.

On the basis of medical knowledge regarding vital signs’ patterns and variabilities (Multimedia Appendix 1), we extracted the frequency spectrums’ positions and values of the local maxima and local minima as the ICU mortality predicting features. Formally, we extracted (1) the value of the frequencies where the oscillations, 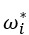, occur; and (2) their corresponding amplitudes, 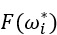, as predictive features (see examples in Figure 2). Specifically, the relative extrema, (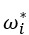, 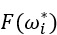) is the local maximum (or local minimum). Namely, 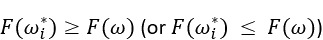 for all values of ω within a threshold distance ε on the frequency spectrum, where ε is a small positive value. We extracted 1 relative extrema point ω^*^ within each distance range (–ε, ε). It should be noted that there are multiple ω^*^ on the entire frequency spectrum, 
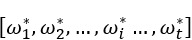
, where *t* is the number of extrema. After we found all relative extrema, *F*(ω^*^), satisfying the requirement, we obtained a vector 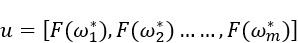. The top *n* maxima are defined as the largest *n* values on *u* (accordingly, the top *n* minima are defined as the smallest *n* values on *u*). When there were <*n* elements in *u*, we adopted all available relative extrema (ie, *m* in total) as features and included *n* – *m* missing values (see parameter selection in Table S1 in Multimedia Appendix 2).

#### Power in Band

**Figure 3:**
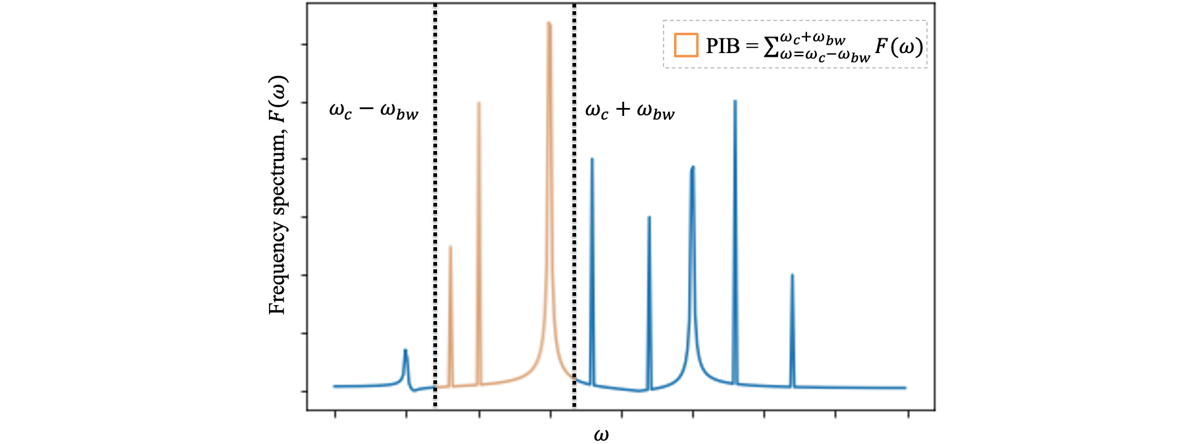
An example of the power-in-band (PIB) feature of the frequency spectrum.

The power-in-band feature is the sum of the total power (see Multimedia Appendix 1 for more detail) within a frequency band (ie, frequency range). With a specified center frequency ω*_c_* and bandwidth ω*_bw_*, we can derive the low and high bounds, ω*_c_* – ω*_bw_* and ω*_c_* + ω*_bw_*, respectively, of the frequency band. The power-in-band feature is 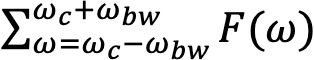 (Figure 3). The power in band summarizes the strength of the signal in the frequency band by computing a single number. The benefits of using power-in-band features are 2-fold. First, the power-in-band feature summarizes the contribution of the given frequency band to the overall strength of the signal, which contains important information regarding vital signs’ stabilities, which summary statistics may not be able to capture (see examples in Multimedia Appendix 1). Second, power in band is a simple yet powerful dimension reduction method for ICU mortality prediction. In practice, we computed the summation (ie, a number) over the different segments of a vector [*F*(ω_1_), *F*(ω_2_),..., *F*(ω*_t_*)] (ie, the vector represents the frequency spectrums transformed from a vital sign; see parameter selection in Table S2 in Multimedia Appendix 2).

#### Statistical Features

Summary statistics are also used to outline and provide information on patients’ health digital traces. For example, the mean of a signal is an estimate of the center of the entire signal. The SD and variance measure the spread extent of the signal from its average value. Taking a patient’s heart rate as a simple example, a normal adult resting heart rate is between 60 and 100 beats per minute. Hence, the mean of the normal heart rate should also be within this range, and the SD should be <7. Abnormal heart rates can be an indicator of a deteriorating health condition. In this study, we calculated various statistic measures of ICU patients’ vital signs as features for ICU mortality prediction (Multimedia Appendix 3).

#### Extreme Values of Moving Windows

**Figure 4:**
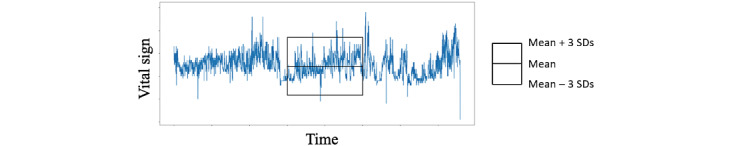
Example of the moving window on the time series of vital signs.

The extreme values of the time series of vital signs over a given period usually indicate unfavorable health conditions as well. We propose a new predictive variable to detect the extreme values on the time-series data. We first created a series of moving windows, and each window had *k* observations. With the *k* observations, we calculated the mean and SD (Figure 4). The observations that were not within 3 SDs of the mean were treated as extreme values [33]. Intuitively, the observations above and below the 3 SDs can be considered as a sudden rise and sudden drop in the vital signs, respectively, both of which have direct relations with patients’ adverse outcomes [33]. We then took the *top* *n* extrema from the moving windows of vital signs, denoted as 
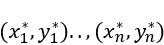
, where x^*^ is the event time and y^*^ is the value of the extrema. When the vital sign in the moving window had <*n* relative extrema (ie, <*n* data points were above and below the 3 SDs of the data in a given moving window), we set all available extreme points (ie, *m* data points) as features and included *n* – *m* missing values (parameter selection can be found in Multimedia Appendix 2).

### ICU Mortality Prediction Using ν-SVC

We defined the mortality prediction as a probabilistic classification problem 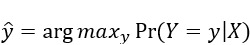. *X* denotes the input space, where




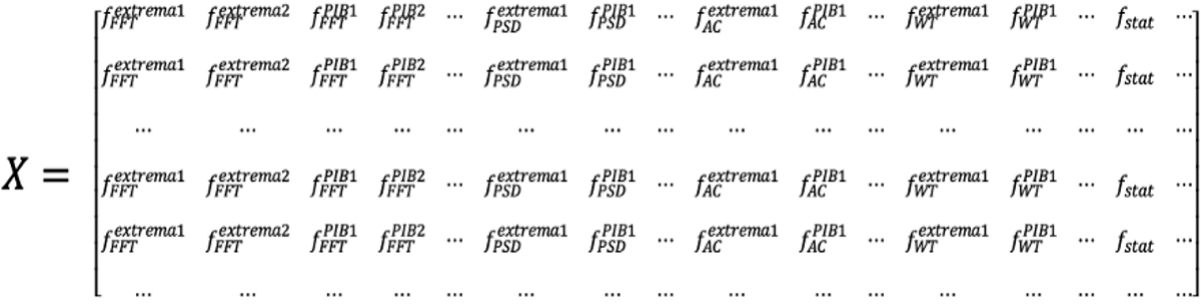




The input space *X* includes features obtained from the previous steps. The output space is defined as *Y* = {1: *expired*, 0: *alive*} (ie, patient ICU discharge status). Our objective was to use a machine learning classifier to establish a mapping function, denoted as *f*(*x*), that effectively maps the input data *X* to the output space *Y*. This mapping function will generate ICU mortality prediction results, represented as *Pr*(*Y*|*X*), where *Pr* is the probability.

For our purposes, we used ν-SVC [34]. ν-SVC learns a maximum-margin decision function in kernel space while regulating model complexity through a single hyperparameter ν. The value of ν simultaneously (1) sets an upper bound on the proportion of training points permitted to lie inside or beyond the margin and (2) sets a lower bound on the proportion of support vectors that define the classifier. Thus, ν offers a direct, interpretable handle on the trade-off between training error and model sparsity without altering the underlying convex quadratic program. Among the multitude of machine learning prediction models, we opted for ν-SVC for 3 key reasons. The first is optimal prediction performance. ν-SVC retains the benefits of other SVC methods, frequently delivering superior performance across various applications. In our specific use case—predicting outcomes in the ICU—prediction performance is of utmost importance. The second reason is handling outliers effectively. Given the heterogeneity of ICU patient cohorts, outliers are common and can significantly impact the prediction results. ν-SVC adjusts the number of support vectors and the margin width based on data characteristics, making it more robust against outliers. The third reason is that it is better equipped to handle imbalanced datasets. In scenarios in which one class significantly outnumbers the others—a situation commonly observed in ICU patient cohorts (Table 3)—ν-SVC is adept at preventing overfitting and bias toward the majority class.

**Table 3:** Dataset description.

		Overall^a^, mean (range)	Missing or unknown (%)	Deceased patients, mean (range)	Alive patients, mean (range)
ICU^b^ stay (h)		85.82 (4.03 to 5925.70)	—^c^	113.61 (4.06 to 5925.70)	83.18 (4.03 to 1987.93)
Age (y)^d^		68.20 (18 to 90)	0.01	71.30 (18 to 90)	67.90 (18 to 90)
**Sex, n (%)**			0.01		
	Male	50.09	—	51.83	50.08
	Female	49.90	—	48.17	49.91
**Ethnicity, n (%)**			5.3		
	African American	11.33	—	9.03	11.54
	Asian	1.51	—	1.71	1.49
	Hispanic	4.16	—	3.35	4.24
	White	77.70	—	80.51	77.44
Height (cm)^e^		168.27 (101.60 to 218.00)	—	168.25 (118.00 to 200.7)	168.027 (101.60 to 218.00)
Weight (kg)		83.43 (22.70 to 295.10)	—	80.58 (27.21 to 275.00)	83.69 (22.70 to 295.10)
**Health digital traces: vital signs**					
	SaO_2_^f^ (%)	96.38 (0 to 100)	0.73	95.56 (0 to 100)	96.48 (0 to 100)
	Heart rate (bpm^g^)	88.22 (0 to 300)	0.02	92.68 (0 to 271)	87.64 (0 to 300)
	Respiration (breaths per min)^h^	21.44 (0 to 200)	6.04	22.47 (0 to 194)	21.30 (0 to 200)
	ST1^i^	0.98 (−20.70 to –700)	48.67	1.20 (−17.00 to –470)	0.95 (−20.70 to –700)
	ST2^j^	1.37 (−14.20 to –830)	47.11	1.90 (−14.15 to –530)	1.31 (−14.20 to –830)
	ST3^k^	1.24 (−24.75 to –1040)	50.62	1.51 (−24.75 to –840)	1.21 (−18.60 to –1040)

^a^The data records are from patients whose admission diagnoses were heart failure (HF), pulmonary sepsis, or renal sepsis. There were 17,025 total admissions (n=5282, 31.02% for HF; n=7308, 42.93% for pulmonary sepsis; and n=4435, 26.05% for renal sepsis). All vital signs used are taken directly from the electronic intensive care unit Collaborative Research Database Vital Periodic table.

^b^ICU: intensive care unit.

^c^Not applicable.

^d^The variable age in the electronic ICU dataset was set to >89 if the patients were aged >89 years. To calculate the mean, we set the ages of the patients aged >89 years to 90.

^e^When calculating the demographic statistics, we removed 11 records with irregular height (eg, 772 cm) or irregular weight (eg, 974 kg).

^f^SaO_2_: arterial oxygen saturation.

^g^bpm: beats per minute; the number of times the heart beats per minute.

^h^The number of breaths a person takes per minute.

^i^ST1: estimated ST segment level 1 of the electrocardiogram (ECG).

^j^ST2: estimated ST segment level 2 of the ECG.

^k^ST3: estimated ST segment level 3 of the ECG.

We chose ν-SVC over deep learning models for 2 reasons. The first is constraints on data availability. In health predictive analysis, access to large volumes of high-quality training data from health care IT systems is not always guaranteed. However, supervised deep learning classifiers typically necessitate substantial amounts of such data for optimal performance. The second reason is complexity versus simplicity in model selection. Rudin [35] countered the common assumption that more complex models necessarily yield more accurate results, debunking the notion that a complicated “black box” is essential for optimal predictive performance. This is often a misconception, particularly with structured data possessing meaningful features. In such instances, there is frequently no significant difference in performance between more complex classifiers (eg, deep neural networks) and simpler ones given adequate preprocessing. Leveraging the structured and highly representative features obtained from previous steps, models with pattern recognition abilities such as CNNs or transformers are not necessary for our research problem. Furthermore, our generated features already incorporate the intricate time-series information, making RNN models, including LSTM and GRU, inapplicable in our case. Furthermore, other generative models such as generative adversarial networks or diffusion models are not suitable for our prediction task.

While we deemed ν-SVC a suitable classifier for our research setting, our proposed research design, involving medical knowledge–driven signal processing for feature extraction coupled with machine learning, offers distinct advantages in extracting meaningful input features beyond the confines of ν-SVC; other machine learning methods can also be used.

To select the most representative features, we used feature selection techniques (ie, ν-SVC with *l*1 penalties) before feeding the entire input feature space, denoted as *X_n_*_×_ *_l_*, into the ν-SVC model. *l*1 penalties are beneficial for feature selection as they result in many estimated coefficients being 0, leaving only the most important features with nonzero coefficients. Our goal in selecting these features was to enhance the performance and accuracy of ν-SVC. Formally, for a set of features *S*, the feature selection method finds the optimal subset *s* of *S* by minimizing the loss function min*_s_*_⊆_*_S_*||*Y* – *Pr*(*Y*|*X*, *X* ∈ *s*)||, where ||·|| is the error estimation function. The classification mapping function *Pr* is determined by the feature selection methods. The selected features *X_n_*_×_ *_m_* are the input of the ICU mortality prediction model, where *m*<*l*.

### Ethical Considerations

The eICU databases were deidentified, anonymized, and approved for sharing by the institutional review boards of both Beth Israel Deaconess Medical Center and the Massachusetts Institute of Technology. Data access was granted to an investigator after the completion of a National Institutes of Health course and successful passing of the associated human research participant protection examination. Given that the data are accessible to the public through the eICU Collaborative Research Database, the need for ethics approval and informed consent was waived. The contributing author SW obtained the necessary authorization to access the anonymized dataset and oversaw the meticulous data extraction process.

## Results

### Data Description

The ICU mortality prediction test bed comes from the eICU Collaborative Research Database [2], which includes data records from multiple hospitals across the United States. The proposed method was established on patients’ health digital traces containing the time series of vital signs. The vital signs in the eICU Collaborative Research Database are consistently interfaced from bedside monitors, which are readily available and updated in real time. To reduce the impact of missing values, we mainly considered the following vital signs that were measured for >50% of patients in our dataset: SaO_2_, heart rate, respiration, ST1, ST2, and ST3 (estimated ST segment level *x* of the ECG, where *x* ∈ {1, 2, 3}), as shown in Table 3. SaO_2_ is useful in understanding the oxygen-carrying capacity of hemoglobin. It is particularly important in patients’ care and management because low oxygen saturation can lead to many acute adverse effects on individual organ systems. Heart rate and respiration are indicators of the body’s basic functions. ST1, ST2, and ST3 are estimated ST segment levels of the ECG.

To show the effectiveness and generalizability of the proposed method, we included ICU patients from 3 different patient cohorts in terms of ICU admission diagnoses: patients with congestive heart failure (HF), pulmonary sepsis, and renal sepsis or urinary tract infection (including bladder). The 3 diagnoses were the most prevalent ICU admission diagnoses in the eICU Collaborative Research Database. The total admissions were 17,025 (n=5282, 31.02% for HF; n=7308, 42.93% for pulmonary sepsis; and n=4435, 26.05% for renal sepsis). Due to the imbalance of the dataset (299/5282, 5.66% deceased for HF; 881/7308, 12.05% deceased for pulmonary sepsis; and 278/4435, 6.27% deceased for renal sepsis), we implemented a stratified 5-fold cross-validation for evaluation. The stratified k-fold cross-validation ensured that each fold was representative of the class proportions in the training dataset. In our research setting, it yielded better bias and variance estimates in cases of unequal class proportions. To alleviate the influence of the data imbalance issue during classifier training, we assigned different weights (
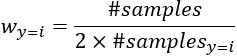
; *i*=1 or 0) to the majority (ie, *Y*={0: *alive*}) and minority (*Y*={0: *expired*}) classes according to the skewed distribution of the classes. The purpose was to penalize minority class misclassification by assigning a greater class weight while decreasing weight for the majority class.

### ICU Mortality Prediction Results

We evaluated the proposed framework for ICU mortality prediction using the first 24-hour time series of health digital traces (this aligns with APACHE IV, which forecasts ICU outcomes 24 hours after admission). We compared our method with four groups of benchmark methods:

1. Severity scoring systems, including APACHE IV [12], the best-performing scoring system that is already used in hospitals
2. Machine learning classifiers with statistical features [18-21]
3. Deep learning models, including 2 CNN models that have previously been used for ICU mortality prediction and have achieved state-of-the-art performance [22,23], as well as LSTM [24] and GRU [36], 2 RNN models that take vital signs in time sequence to estimate mortality rate
4. Time-series forecasting methods, the classic statistical time-series forecasting methods; following previous research [25], we fit the ARMA and ARIMA models on vital signs and took the estimated coefficients as the inputs of machine learning classifiers to predict mortality probabilities (details are available in Multimedia Appendix 2). The results are summarized in Table 4 (parameter specifications can be found in Multimedia Appendix 2).

**Table 4:** Evaluation of the proposed method and baseline methods.

Category and method			AUC^a^		Improvement of our proposed framework over each baseline method (%)^b^
**Severity scoring system**					
	APACHE^c^ IV [12]	0.750		17.6	
**Traditional machine learning model with feature engineering^d^**					
	Decision tree [18]	0.681		29.52	
	Random forest [19]	0.748		17.91	
	Logistic regression [21]	0.749		17.76	
	Gradient boosting [20]	0.775		13.81	
**Deep learning model with raw clinical data**					
	GRU^e^ [36]	0.722		22.16	
	CNN^f^ model 1 [23]	0.732		20.49	
	CNN model 2 [22]	0.712		23.88	
	LSTM^g^ [24]	0.698		26.36	
**Time-series forecasting model**					
	ARMA^h^ coefficients [25]	0.660		33.64	
	ARIMA^i^ coefficients [25]	0.611		44.35	
**Our proposed framework**					
	ν-SVC^j^	0.882		—^k^	

^a^AUC: area under the curve.

^b^Improvement: percentage increase = (AUC_ours_ – AUC_baseline_)/AUC_baseline_.

^c^APACHE: Acute Physiology and Chronic Health Evaluation.

^d^Each of the original studies used multiple machine learning classifiers, and the reported best-performing classifier in the original paper was selected as the benchmark.

^e^GRU: gated recurring unit.

^f^CNN: convolutional neural network.

^g^LSTM: long short-term memory.

^h^ARMA: autoregressive moving average.

^i^ARIMA: autoregressive integrated moving average.

^j^SVC: support vector classification.

^k^Not applicable.

The experiment yielded several findings. First, our method achieved the highest AUC, demonstrating a significant improvement compared to all the baseline methods. Second, our proposed method demonstrated a notable performance improvement of 17.6% compared to APACHE IV, which is the best-performing scoring system in ICU outcome prediction that is already used in hospitals. In contrast to our method, APACHE IV relies on more resource-demanding features for predictions, including laboratory test results (which can be time-consuming to obtain) and intensivists’ assessments (which may not always be available). Our approach exhibited better performance while using fewer resources when compared to the best severity scoring system. Third, compared to the best-performing traditional machine learning model with feature engineering [19] (AUC=0.775), our method improved the AUC by 13.81%. The significant performance improvement achieved indicates the inadequacy of traditional statistical-based feature engineering methods in processing complex ICU vital sign time series and predicting ICU outcomes. Our proposed signal processing techniques, guided by medical knowledge, proved to be highly effective in extracting features and greatly benefited ICU outcome prediction. Fourth, despite the dominance of deep learning models in the field of data science and machine learning, all the deep learning–based baseline methods were significantly outperformed by our proposed method. A possible reason is our explicit extraction of valuable information from the patients’ health digital traces, which facilitated the classifiers’ identification of the relationship between the input space (ie, patients’ health digital traces) and the prediction outcome (ie, ICU mortality), thus improving overall performance.

Another primary objective of this study was to introduce a new method for the effective extraction of patterns from the complex time series of vital signs in patients’ health digital traces. Therefore, we further compared the performance of different feature sets. The experiment yielded the following findings. First, the feature set we proposed included statistical features and signal processing features. We examined their effectiveness. We excluded statistical features and signal processing features and reconducted the evaluation. Using only signal processing features versus only statistical features, the predictive model reached AUCs of 0.828 and 0.749, respectively. The results indicate that both signal processing and statistical techniques can extract informative features and these extracted features are either more predictive than or comparable to the APACHE IV (AUC=0.750) features for ICU mortality prediction. Second, the obtained AUC scores of signal processing features (AUC=0.828) were higher than those of statistical features (AUC=0.749), validating the necessity of using signal processing techniques for decomposing the complex time series of health digital trace data and the necessity of the proposed medical knowledge–guided feature extraction methods. Third, to examine whether the proposed method can add value to existing systems such as APACHE IV, we merged the features generated by our method with other features available in the APACHE IV system. These features include patient demographics and other attributes available at ICU admission, which can have great value for ICU mortality prediction. We intentionally excluded variables that demand laboratory resources (eg, arterial blood gas) or assessments by intensivists (eg, GCS) to maintain resource efficiency in our prediction model. The new feature set attained an AUC of 0.886, demonstrating that the features generated using our method can be effectively incorporated into other ICU mortality prediction models. The subsequent model is expected to display enhanced predictive capability and reduce the demand for time-consuming or resource-intensive human evaluations.

**Figure 5:**
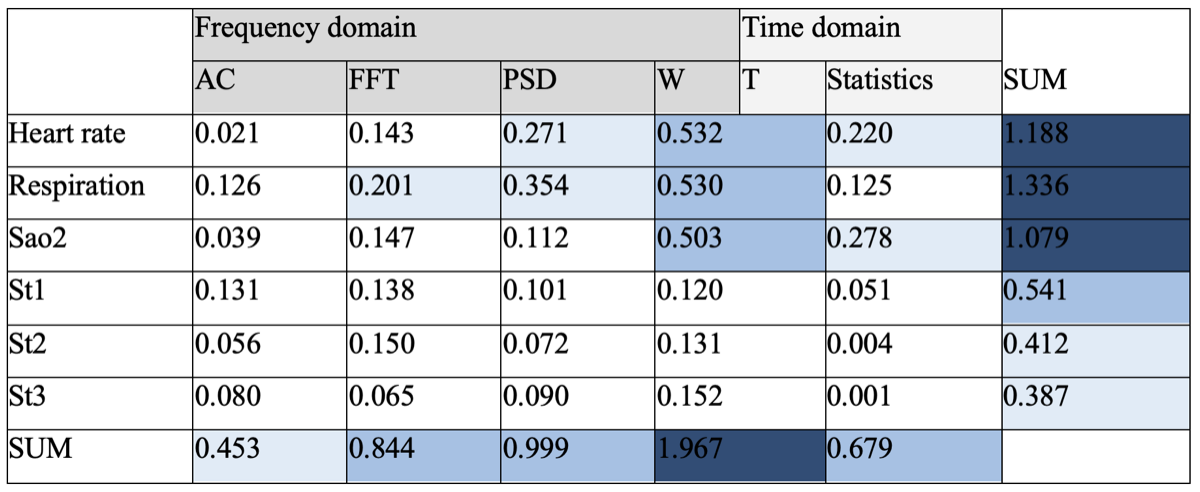
Summary of the importance of different feature types. The cells in the Statistics column contain the sum of feature importance across all statistical features for the corresponding vital sign, including SD, variance, mean, median, quantiles, min, max, and the first and last signal of the vital sign. AC: autocorrelation; FFT: fast Fourier transform; PSD: power spectral density; SaO2: arterial oxygen saturation; ST1: estimated ST segment level 1 of the electrocardiogram (ECG); ST2: estimated ST segment level 2 of the ECG; ST3: estimated ST segment level 3 of the ECG; WT: wavelet transform.

To investigate the contributions of different vital signs and signal processing techniques toward predicting ICU outcomes, we aggregated the feature importance for each group. As shown in Table 3, heart rate, respiration, and SaO_2_ exhibited the highest contribution compared to other vital signs in the mortality prediction task. These 3 vital signs are not difficult or expensive to measure in eICUs. In the eICU Collaborative Research Database, heart rate, respiration, and SaO_2_ were constantly measured for >90% of patients. Furthermore, among all the proposed signal processing techniques, WT yielded the most informative features. A possible reason is that WT can reveal patterns from both the time and frequency domains simultaneously. In addition, autocorrelation provided the least instructive features. As a signal processing technique, autocorrelation is conceptually close to time-series forecasting algorithms such as ARMA and ARIMA. The unsatisfactory performance of autocorrelation reveals the fact that time-series forecasting algorithms can hardly capture sufficient information for ICU mortality prediction. This observation is also supported by our experiment using ARMA and ARIMA coefficients for prediction (Figure 5).

### Postanalysis: Assessing Predictive Features of ICU Mortality

One of the objectives of this work was to extract representative features from the complex time series of vital signs in ICU patients’ health digital traces, which is accomplished by leveraging medical domain knowledge and using signal processing techniques to decompose the time-series data, enhance relevant signals, and minimize noise within the complex time series. In this section, we report on the postanalyses conducted to examine why our proposed method achieved better performance compared to other existing methods.

We first selected and compared 2 patients from our dataset as an illustrative example to demonstrate the feasibility of signal processing techniques and our proposed feature extraction methods in ICU outcome prediction (Table 5). According to medical knowledge, the worst vital sign values and fluctuating vital signs all have direct relations with adverse ICU outcomes [12,37]. The health digital traces’ fluctuating patterns of the 2 patients were distinct, and it was not intuitive to predict their ICU outcome based on their health digital traces (Table 5, Health digital traces—time series of vital signs). The SaO_2_ of patient 1 dropped to 58% (the lowest value) and fluctuated at the beginning of her ICU stay. Her SaO_2_ stabilized at approximately 840 minutes and stayed at 100%. The lowest SaO_2_ value of patient 2 (80%) was better than that of patient 1. However, her SaO_2_ did not stabilize during her 24-hour stay in the ICU as it continued to fluctuate.

**Table 5:** Illustrative example demonstrating the feasibility of the proposed feature sets.

			Patient 1 (outcome: alive)		Patient 2 (outcome: deceased)
Health digital traces—time series of vital signs			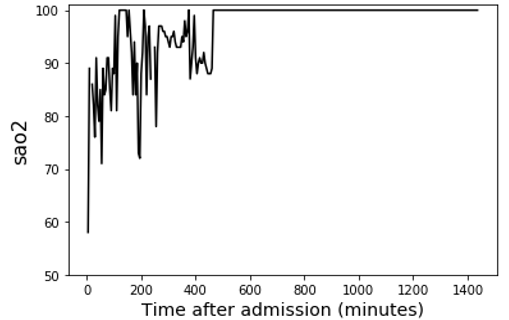		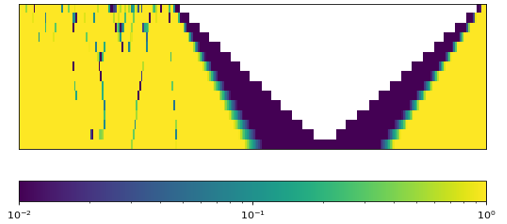
Time-series decomposition using signal processing techniques (using wavelet transform as an example)			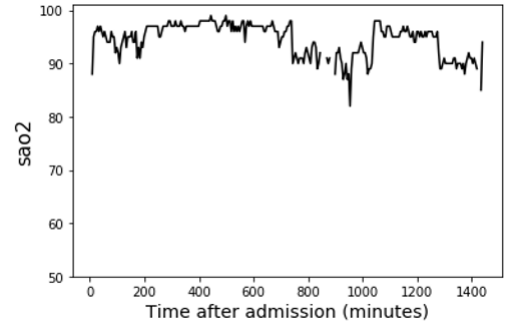		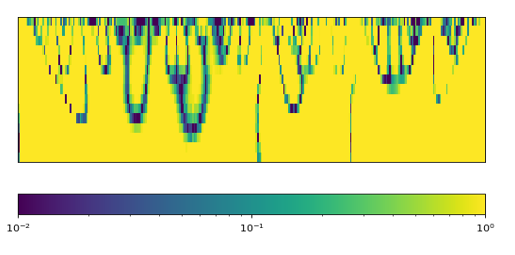
			Shows distinct differences between the stable and unstable time series of SaO_2_^a^.		Shows unstable SaO_2_ during the entire ICU^b^ stay. Unstable vital signs have a direct relation with adverse ICU outcomes.
**Example from the proposed feature set**					
	sao2_sudden-drop_value_1^c^	58% (less sudden changes)		82% (more sudden changes)	
	sao2_wt_morl_length5_power-in-band_1^d^ (power-in-band feature; band_1: low-frequency band. A higher value of this feature indicates that the smooth part [ie, no fluctuation, indicating favorable health condition] of the vital sign is longer and the value of the smooth part of the vital sign is higher [ie, high SaO_2_, indicating a favorable health condition])	13,378.21335		8985.535909	
	stat_sao2_last^e^	100 (the last value at the time of prediction is within the normal range)		85 (the last value at the time of prediction is NOT within the normal range)	
	Other features	There are many other features not included in this table as cases.		There are many other features not included in this table as cases.	
**Example from the APACHE^f^** **IV feature set**					
	Age (y)	79		83	
	Gender	Female		Female	
	Has active treatment	Yes		Yes	
	Has diabetes (diabetes is a chronic condition that may lead to adverse ICU outcomes)	Yes		No	
	GCS^g^ score (depends on expert assessments; the higher the better)	6		15	
	Other features	There are many other features not included in this table as cases.		There are many other features not included in this table as cases.	
**Example from the statistical feature set**					
	stat_sao2_min^h^ (the worst [minimum] value of the vital sign; the higher the SaO_2_ the better)	58		82	
	stat_sao2_std^i^ (the fluctuation [SD] of the vital sign; the lower the better)	6.05375		3.20926	
	Other features	There are many other features not included in this table as cases.		There are many other features not included in this table as cases.	
**Death probability at 24 h**					
	APACHE IV	86.4% (ICU mortality prediction result was wrong)		8.3%	
	Our method	31.8%		59.8% (ICU mortality prediction result was correct)	

^a^SaO_2_: arterial oxygen saturation.

^b^ICU: intensive care unit.

^c^sao2_sudden-drop_value_1: vital sign signal: SaO_2_; feature extraction: extreme values within moving windows (sudden drops with an SD of 1).

^d^sao2_wt_morl_length5_power-in-band_1: vital sign signal: SaO_2_; signal processing: wavelet transform using Morlet wavelets. Feature extraction: power in band (segment length of 5 and band_1).

^e^stat_sao2_last: vital sign signal: SaO_2_. Signal processing: statistical feature (the last value of the vital sign).

^f^APACHE: Acute Physiology and Chronic Health Evaluation.

^g^GCS: Glasgow Coma Scale.

^h^stat_sao2_min: the worst value of the vital sign.

^i^stat_sao2_std: the amount of variation in SaO_2_.

The illustrative example reveals several important observations related to predictive features of ICU outcome predictions. First, APACHE IV only included the vital signs’ worst measurements in the first 24 hours of ICU stay. For patient 1, it ignored the important healthy signal that the second half of the SaO_2_ conveys, which caused APACHE IV to make a wrong inference (86.4% death probability, but patient 1 was alive at the time of discharge). In addition, APACHE IV did not capture the vital sign fluctuation of patient 2 and made a wrong prediction (8.3% death probability, but she died later). Moreover, the GCS score is a variable depending on expert evaluation in APACHE IV (the higher the better; 3 being the worst and 15 being the best). Patient 1’s GCS score was lower than that of patient 2, but patient 1 survived, demonstrating that such a predictor is not always indicative of ICU outcomes. Second, in extant studies, researchers consider simple statistical features of vital signs (Table 5, Example from the statistical feature set). The *stat_sao2_min* variable indicates the worst value of the vital sign. The *stat_sao2_std* variable indicates the amount of variation in SaO_2_, which represents the fluctuation in SaO_2_. Both values suggest that patient 2 had a higher likelihood of survival. However, the actual ICU outcome differed from the prediction, showing that existing statistical features are inadequate in capturing complex patterns from ICU patients’ digital traces. Third, the proposed framework can effectively identify meaningful patterns in ICU patients’ health digital traces and lead to better ICU outcome predictions (Table 5, Example from the proposed feature set). The signal processing result of patient 1’s SaO_2_ shows the distinct differences between the stable and unstable time series (Table 5, Signal processing decomposition results). The proposed feature extraction methods, such as *sao2_sudden-drop_value_1* and *sao2_wt_morl_length5_power-in-band_1*, can properly capture the vital sign’s stability information and result in correct prediction of patient outcomes. Overall, the example demonstrates the insufficiency of existing approaches and the motivation to identify more accurate indicators for predicting ICU outcomes. In the meantime, the proposed framework can catch patterns in the time series of vital signs that are difficult to detect using other methods, such as APACHE IV and statistical features. Our method can offer valid feature sets for the prediction of ICU outcomes.

**Figure 6:**
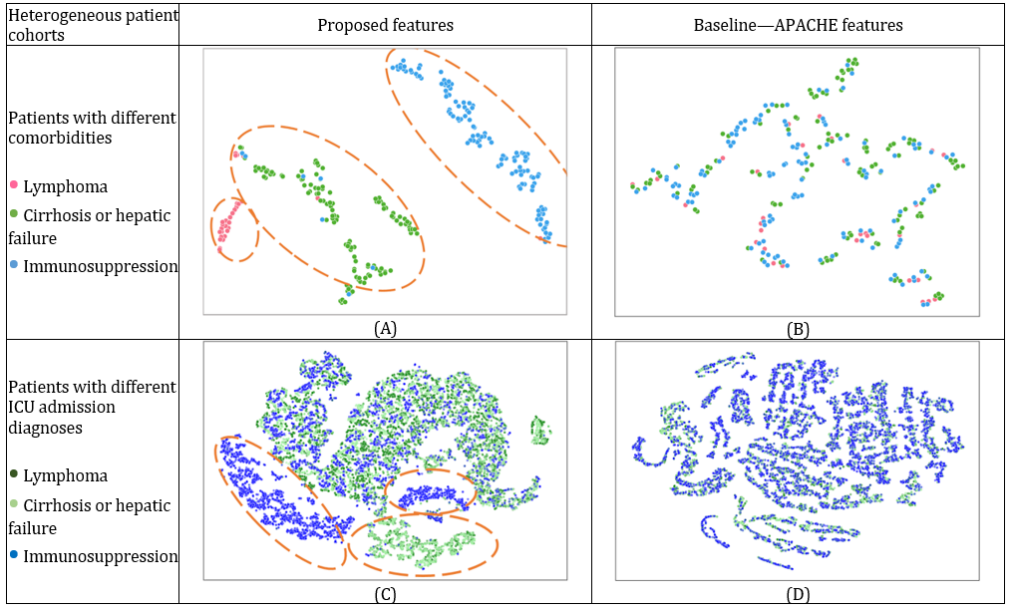
Feature representativeness for heterogeneous intensive care unit (ICU) patient cohorts. APACHE: Acute Physiology and Chronic Health Evaluation; t-SNE: t-distributed stochastic neighbor embedding.

Furthermore, patients from different cohorts exhibited distinct disease progressions. To evaluate the efficacy of the proposed features in representing ICU patients from heterogeneous cohorts, we visualized the proposed feature set and compared the resulting visualizations to those of the APACHE IV feature set. As shown in Figure 6, the proposed feature set can effectively distinguish patients with different comorbidities and patients with different ICU admission diagnoses (Figures 6A and 5C). In contrast, APACHE IV features were not able to discriminate between patients from different cohorts (Figures 6B and 5D). According to medical literature, different patient cohorts typically experience varying disease progression and outcomes [7]. Therefore, our method, which has strong capabilities to extract patterns and represent ICU patients from heterogeneous cohorts, can facilitate ICU outcome prediction (as we demonstrate in the ICU Mortality Prediction Results section).

### Postanalysis: The Impact of Limited Patient Data on Deep Learning Model Performance

Deep learning models exhibit superior performance due to their reliance on substantial computing power, advanced algorithmic capabilities, and extensive training data, enabling them to yield highly promising results across diverse domains. Nonetheless, patient data in health care predictive analysis, including ICU outcome prediction, are highly specific. ICU patients come from various diagnostic cohorts, have unique demographics and disease progressions, and may receive different levels of medical intervention. The integration of patient data must be executed with great care, making it impractical to acquire sufficient training data for complex deep learning models.

The benchmark methods used were the best-performing methods within each category, as outlined in Table 4.

**Figure 7:**
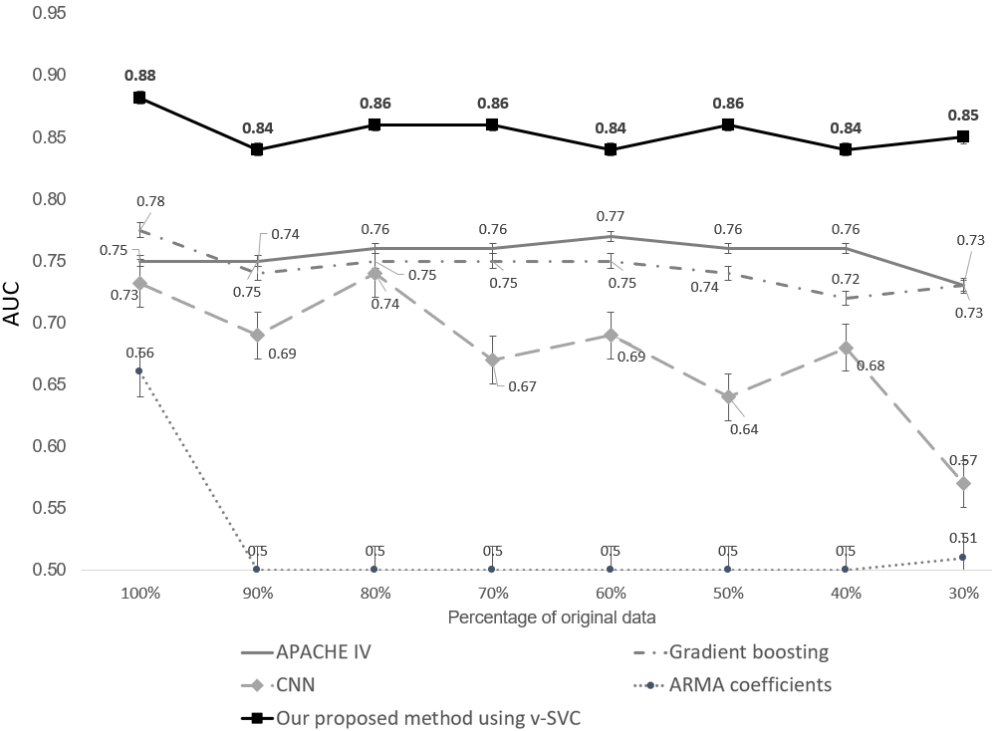
Evaluation of the proposed method and baseline methods. APACHE: Acute Physiology and Chronic Health Evaluation; ARMA: autoregressive moving average; AUC: area under the curve; CNN: convolutional neural network; SVC: support vector classification.

In our experimental evaluation, we chose to focus on the 3 most prevalent patient cohorts (ICU admission diagnoses) within the eICU Collaborative Research Database. The number of patients per cohort had already reached the upper limit in this database (5282/17,025, 31.02% for HF; 7308/17,025, 42.93% for pulmonary sepsis; and 4435/17,025, 26.05% for renal sepsis), with other patient cohorts containing fewer patients. To our knowledge, the eICU Collaborative Research Database is already one of the largest publicly available ICU databases. In contrast, in other problem domains, such as natural language processing or image processing, the training data for deep learning models typically extend into the millions or even billions. In the aforementioned 3 patient cohorts, when using the complete dataset, deep learning models did not outperform our approach. To explore the limitations and boundaries further, we systematically reduced the original dataset (to 90%, 80%, 70%, 60%, 50%, 40%, and 30%) to observe the performance variations of our proposed method and the benchmark methods, as illustrated in Figure 7 [12,19,21,23]. The results showed that (1) our proposed method consistently outperformed all benchmark methods, (2) traditional machine learning approaches with feature engineering and scoring systems also demonstrated better performance compared to deep learning methods, and (3) deep learning methods failed to converge when 30% of the original data were available.

Moreover, we conducted tests on our proposed method and the benchmark methods using 3 small patient cohorts (in terms of ICU admission diagnosis). It was noted that obtaining satisfactory performance results using deep learning methods posed a challenge for these smaller patient cohorts. Nevertheless, our approach (as well as other traditional machine learning methods with feature extraction) exhibited stable performance over various patient cohorts (Table 6). The model parameters are listed in Table S3 in Multimedia Appendix 2.

**Table 6:** Evaluation of the proposed method and baseline methods—3 small patient cohorts.

Category and method			ICU^a^ admission diagnosis, AUC^b^				
			Atelectasis (36 deceased vs 309 alive)		Pneumothorax (14 deceased vs 295 alive)		Cardiomyopathy (29 deceased vs 539 alive)
**Severity scoring system**							
	APACHE^c^ IV [12]	0.642		0.774		0.812	
**Traditional machine learning model with feature engineering**							
	Decision tree [18]	0.951		0.824		0.923	
	Random forest [19]	0.889		0.852		0.944	
	Logistic regression [21]	0.851		0.764		0.891	
	Gradient boosting [20]	0.932		0.831		0.832	
**Deep learning model with raw clinical data**							
	GRU^d^ [36]	0.602		0.889		0.694	
	CNN^e^ model 1 [23]	0.671		0.842		0.584	
	CNN model 2 [22]	0.620		0.561		0.589	
	LSTM^f^ [24]	0.641		0.532		0.621	
**Time-series forecasting model**							
	ARMA^g^ coefficients [25]	0.502		0.510		0.521	
	ARIMA^h^ coefficients [25]	0.501		0.521		0.501	
**Our proposed method**							
	ν-SVC^i^	0.978		0.989		0.981	

^a^ICU: intensive care unit.

^b^AUC: area under the curve.

^c^APACHE: Acute Physiology and Chronic Health Evaluation.

^d^GRU: gated recurring unit.

^e^CNN: convolutional neural network.

^f^LSTM: long short-term memory.

^g^ARMA: autoregressive moving average.

^h^ARIMA: autoregressive integrated moving average.

^i^SVC: support vector classification.

The aforementioned 2 experiments illustrate that, in health care predictive analysis, particularly in ICU outcome prediction, when acquiring a large amount of training data is not feasible, the limitations of deep learning models become evident. In contrast, our proposed research design, which involves medical knowledge–driven feature extraction coupled with machine learning, holds significant potential owing to its efficiency.

## Discussion

### Principal Findings

ICU patients’ health digital traces contain complex time-series data and patterns. It is essential to find representative features to develop predictive models for better ICU outcome predictions. Guided by signal processing techniques and medical domain knowledge, we propose a novel method to repurpose and effectively extract features with strong predictive power from patients’ health digital traces for ICU mortality prediction. We systematically evaluated the proposed method using a real-world multicenter ICU database from the perspective of feature effectiveness and prediction accuracy. The proposed method efficiently extracted representative features from heterogeneous patient cohorts. The ICU outcome prediction results significantly outperformed those of state-of-the-art benchmarks.

Our contribution lies in incorporating medical knowledge to guide the selection of the most suitable signal processing techniques and feature extraction methods for predicting ICU outcomes. Our approach presents generalizable design principles for research scenarios with limited training data, demonstrating how integrating domain knowledge into signal processing and predictive model design enhances performance. Our work has important implications for health care operation management. We contribute to the emerging field of using digital traces from information systems to address challenges with significant implications for health care [1]. Specifically, we present a new feature extraction method that uses patients’ digital traces retrieved from health IT systems to predict ICU mortality accurately. Practically, accurate prediction of ICU outcomes is important. It indicates when patients may require heightened attention, care, and interventions; therefore, all essential resources, including personnel, equipment, and medications, are readily available to ensure the provision of comprehensive support for the patient.

While the results are encouraging, the proposed method is not without limitations. First, more vital sign data (eg, the central venous pressure and pulmonary artery pressure) were not included due to the high missing rate. The predicting power of these vital sign data can be evaluated in the future. Next, our method could be enhanced by developing a more comprehensive model that incorporates individual patient characteristics such as medical history and genetic information. By doing so, our method holds the potential to evolve into a generalized mortality prediction model tailored to each patient’s unique profile.

### Conclusions

To conclude, as the Fourth Industrial Revolution evolves, digital tools create an influx of data. In health care, this trend has transformed ICUs by enabling the collection of real-time patient health data, leading to critical advances in ICU outcome predictions—a task with high stakes due to patients’ rapid disease progression and high mortality rates. The availability of digital health data provides new opportunities to refine these prediction models. This study created a new feature extraction method that aims to enhance the accuracy of ICU outcome predictions by repurposing digital trace data from ICU patients. The resulting method outperformed existing ICU outcome prediction models. Our study has important implications for health care operation management by using digital traces from health care information systems to solve problems with societal implications and leveraging specific domain knowledge to create innovative and impactful artifacts. Practically, the proposed method efficiently extracted representative features, facilitating ICU outcome prediction.

## Appendix

Multimedia Appendix 1 Medical domain knowledge–guided feature extraction.

Multimedia Appendix 2 Experiment setup and parameter selection.

Multimedia Appendix 3 Statistical features and relations to health digital traces.

## Abbreviations

APACHE: Acute Physiology and Chronic Health Evaluation
ARIMA: autoregressive integrated moving average
ARMA: autoregressive moving average
AUC: area under the curve
CNN: convolutional neural network
ECG: electrocardiogram
eICU: electronic intensive care unit
FFT: fast Fourier transform
GCS: Glasgow Coma Scale
GRU: gated recurrent unit
HF: heart failure
ICU: intensive care unit
LSTM: long short-term memory
PSD: power spectral density
RNN: recurrent neural network
SaO2: arterial oxygen saturation
SVC: support vector classification
WT: wavelet transform
