# Supplementary material for: Intensive Care Unit Patient Outcome Prediction Using ν-Support Vector Classification and Stochastic Signal Processing–Based Feature Extraction Techniques: Algorithm Development and Validation Study
Source: JMIR AI. 2025 Aug 26;4:e72671. doi: 10.2196/72671 (PMC12421204; doi:10.2196/72671)
Supplement: Multimedia Appendix 1 [file ai_v4i1e72671_app1.docx]

**Multimedia Appendix 1.** Medical domain knowledge–guided feature extraction.

| Medical guidelines and motivation^a^ | | | | Proposed feature extraction methods |
| --- | --- | --- | --- | --- |
| Typical patterns in health digital traces^b^ | | | Relation to the time series (ie, time domain) and decomposed time series (ie, frequency domain) of health digital traces |  |
|  | | | | |
| **Vital sign normal range—vital signs have normal ranges (the normal values vary by patients’ heterogeneous conditions); sets of vital signs outside normal ranges typically indicate the need for interventions** | | | | |
|  | 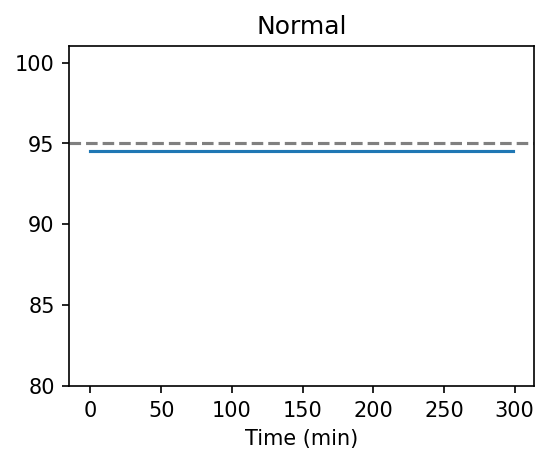  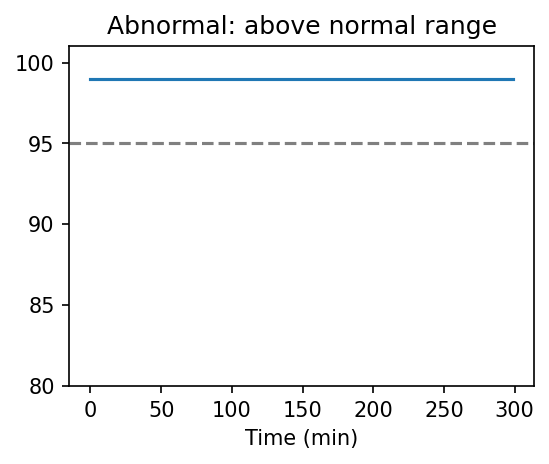  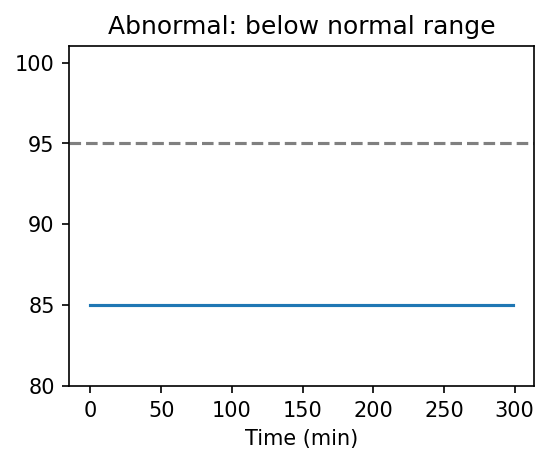 | | - Time domain: measures the central tendency of vital signs. Values outside normal ranges indicate a patient’s deteriorating condition. | Mean (*Vt*); median (*Vt*); quantiles (*Vt*) |
| **Vital sign best or worst measurement value—determine patient condition using the best or worst vital sign measurement value** | | | | |
|  | 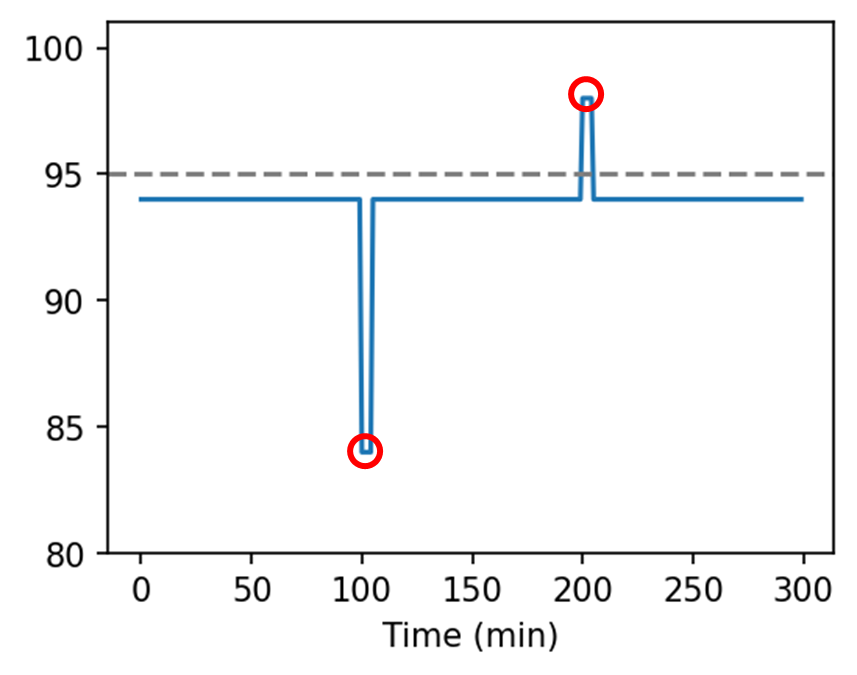 | | - Time domain: extract the extreme values as predictive variables. | Minimum (*Vt*); maximum (*Vt*) |
| **Vital signs upon arrival at the ICU^c—^determine patient condition using the vital signs upon arrival at the ICU** | | | | |
|  | 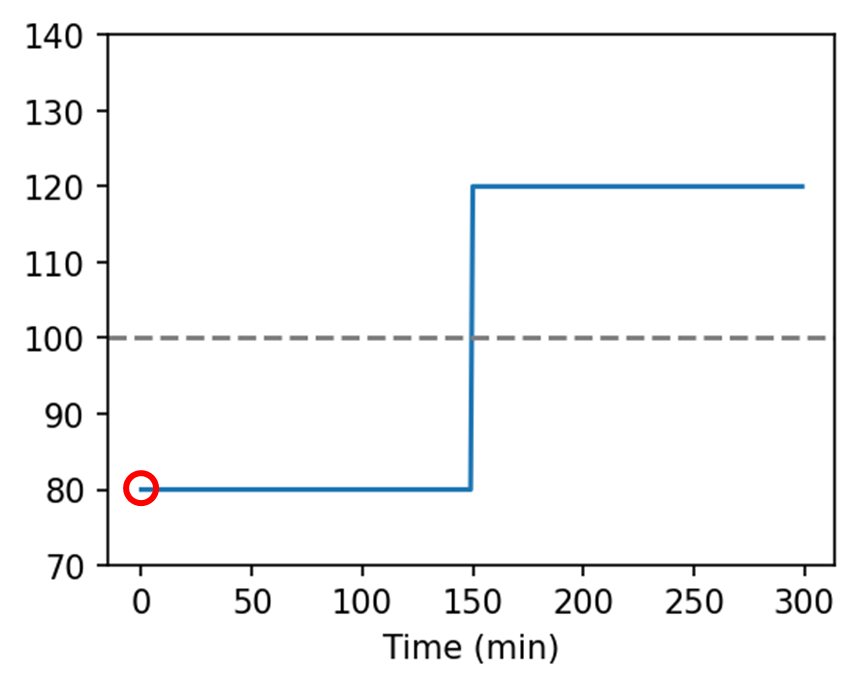 | | - Time domain: extract the first value of a vital sign as the predictive variable. | First: *V*_1_ |
| **Vital signs at the time of prediction—determine patient condition using the latest vital sign measurements at the time of outcome prediction** | | | | |
|  | 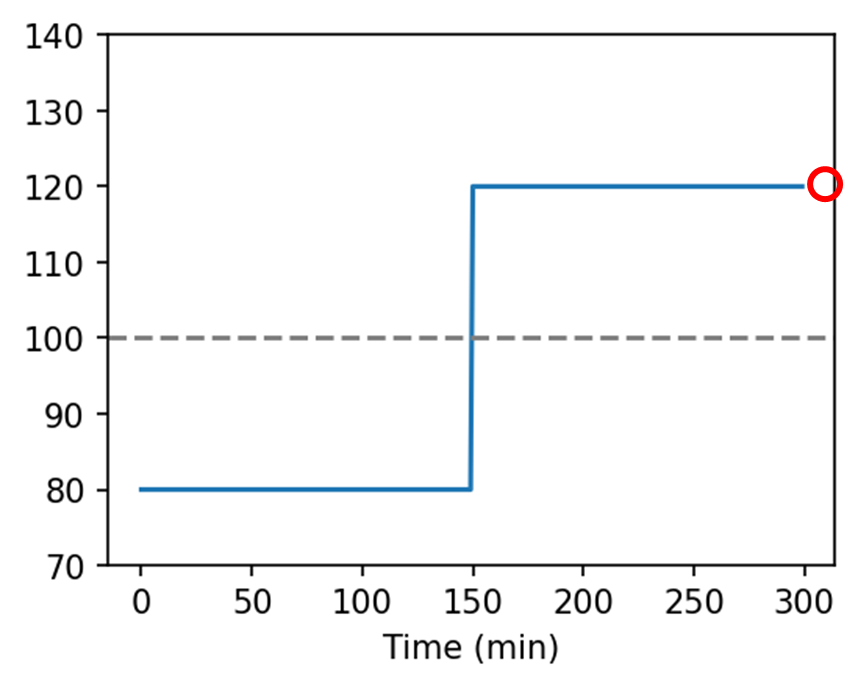 | | - Time domain: extract the last value of a vital sign as the predictive variable. | Last: *V_N_* |
| **Stability of vital signs—unstable vital signs are associated with clinical risks** | | | | |
|  | **Overall stability^d^** | | | |
|  |  | 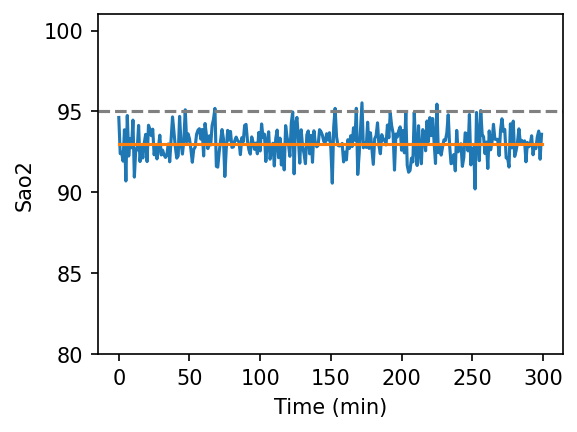 | - Time domain: measure the amount of variation or dispersion of vital signs | SD: $\sqrt{Var(V)}$; variance: Var(*V*) |
|  |  | 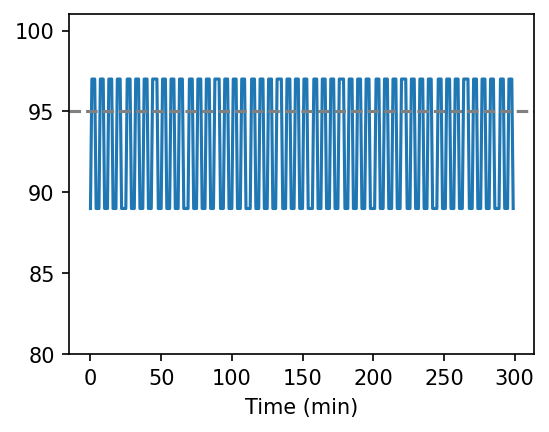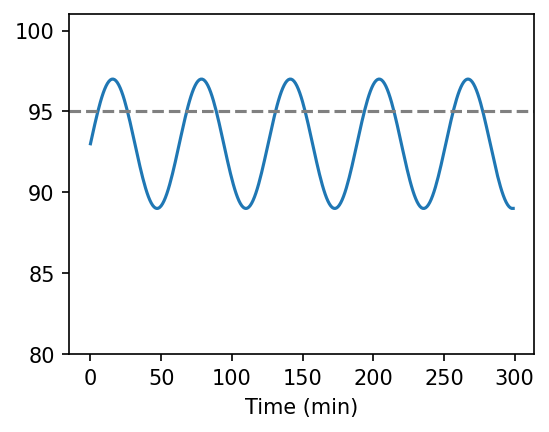 | - Frequency domain: capture the overall stability of vital signs that summary statistics cannot capture.   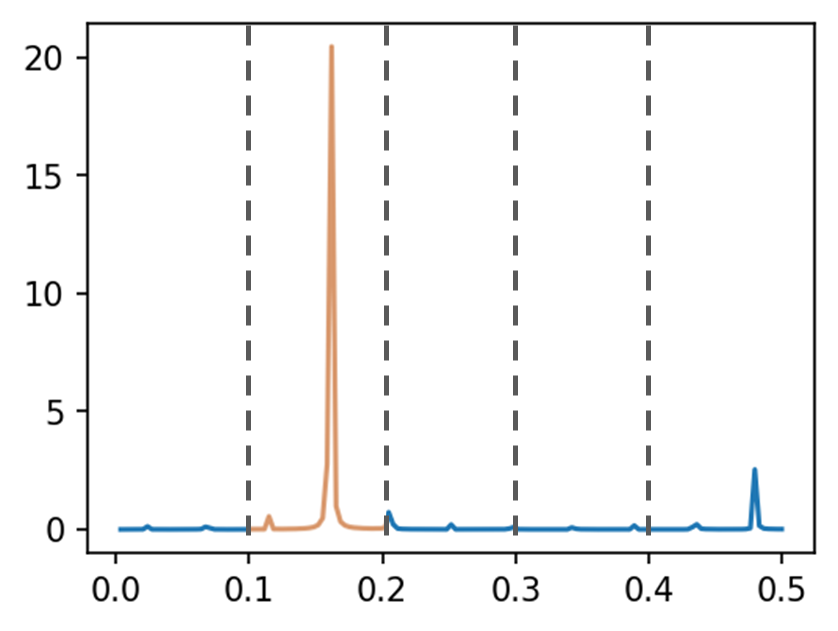  (band_1, band_2,..., band_5)   - Dramatic fluctuation: unfavorable health conditions—large values in the higher-frequency band (eg, band_2).   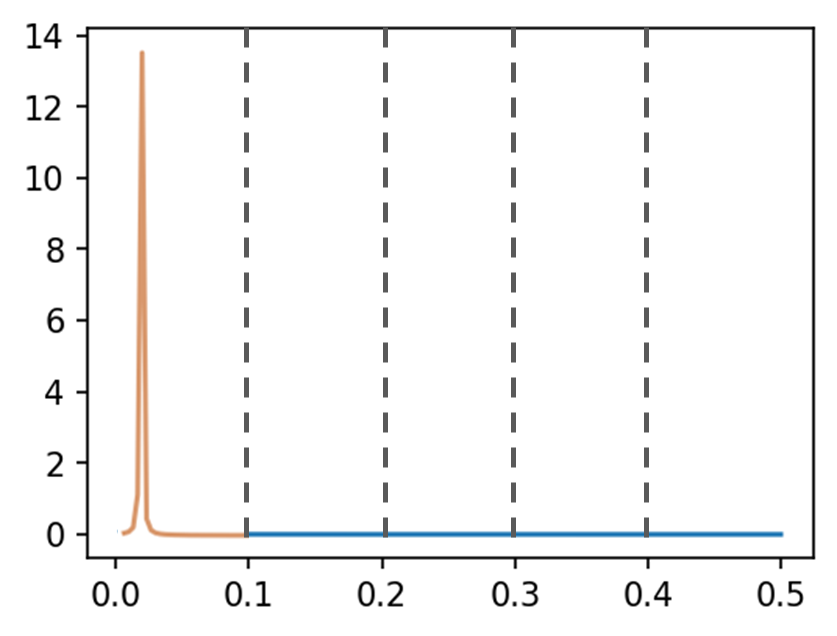  (band_1, band_2,..., band_5)   - Smooth curve: favorable health conditions—large values in the lower-frequency band (eg, band_1). | Power in band |
|  | **Sudden change^e^** | | | |
|  |  | 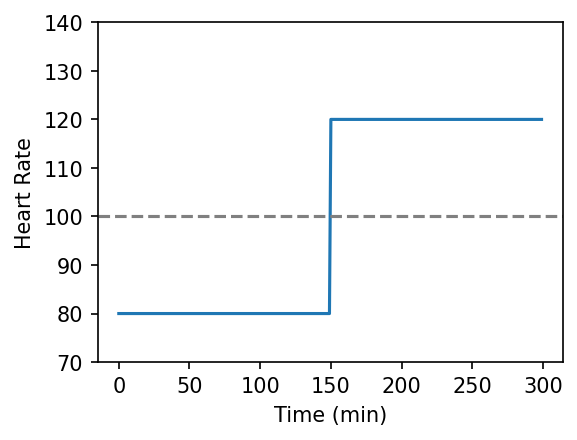 | 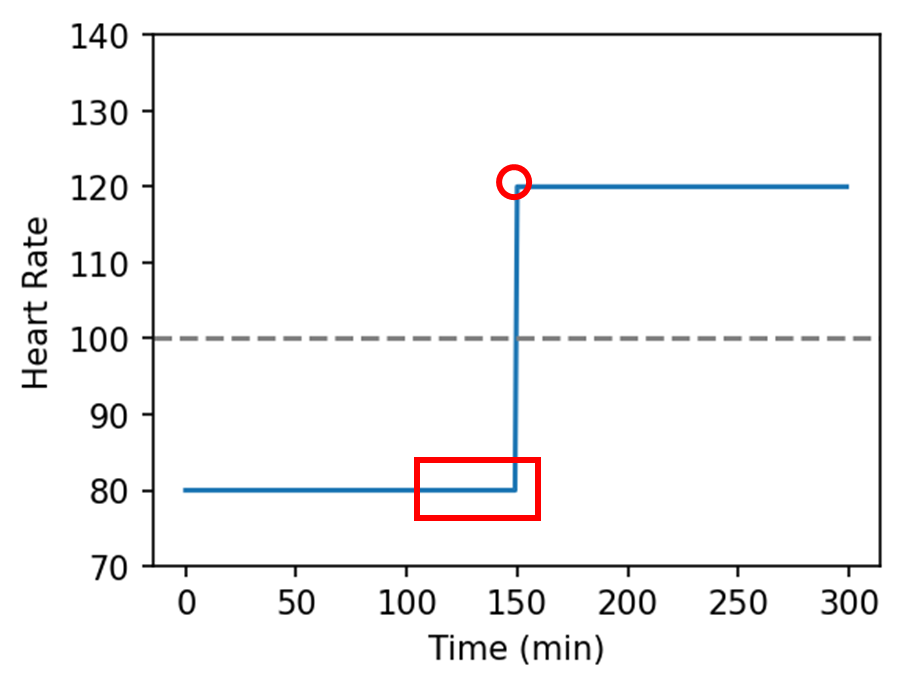   - Time domain: capture the recency and frequency of sudden changes in the time series of vital signs as input variables | Extreme values in moving windows |
|  | **Short-term and long-term variabilities^f^** | | | |
|  |  | 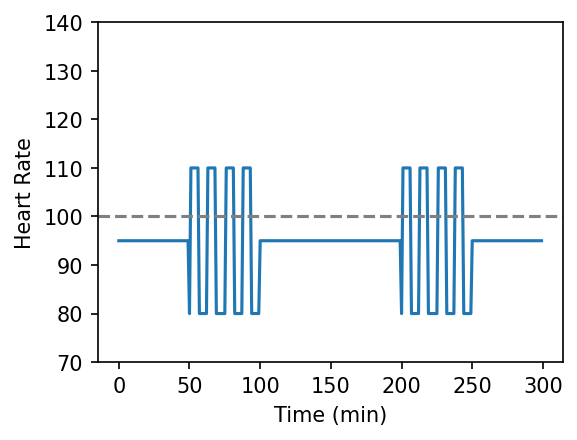  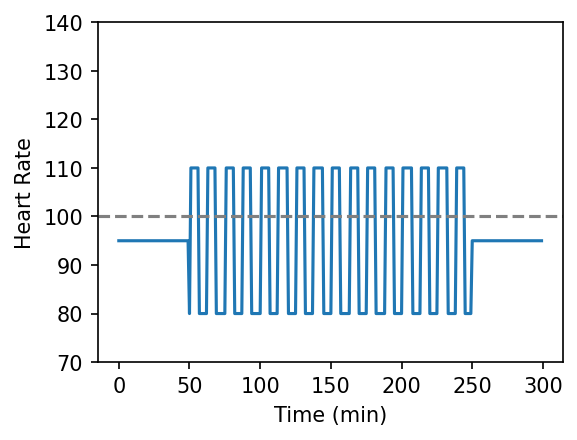 | 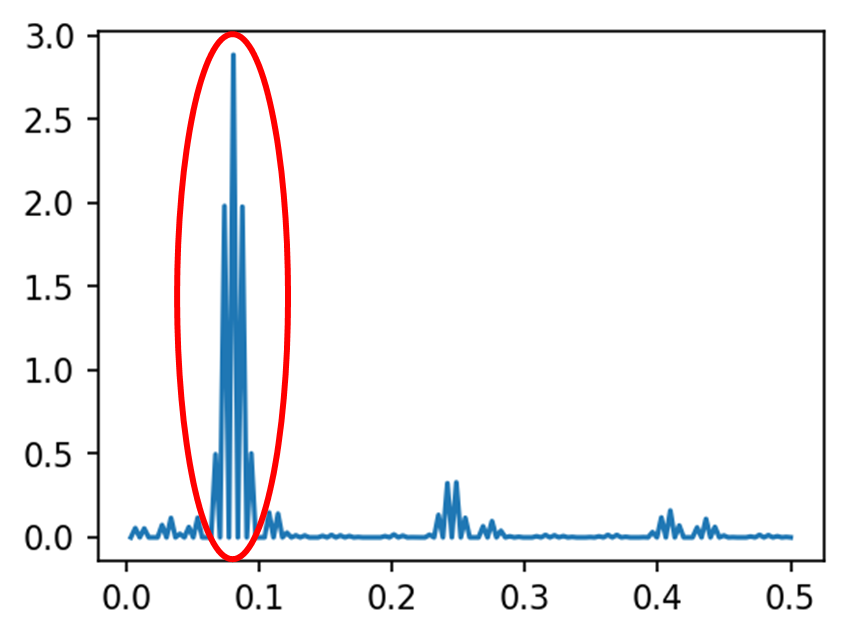  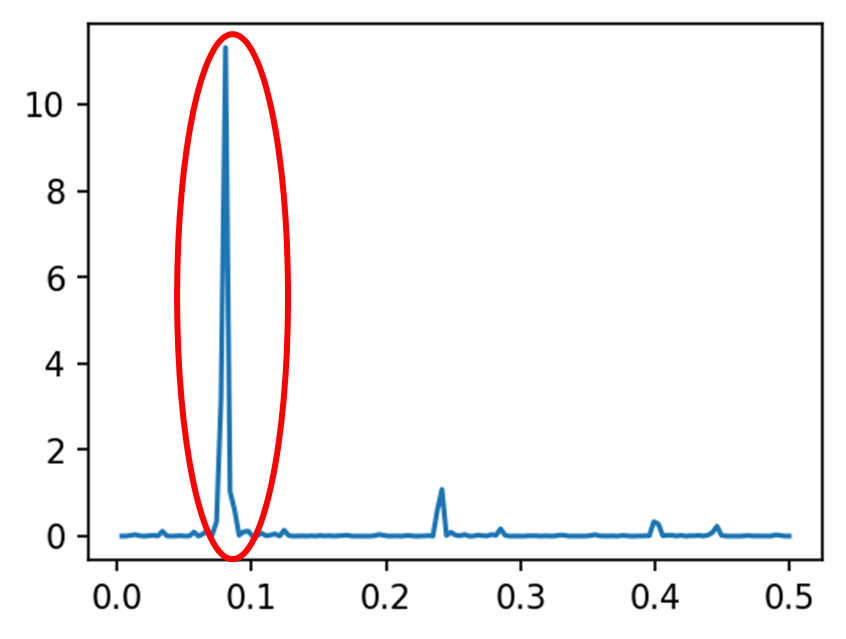   - Frequency domain: combining positions and values of relative extrema can distinguish vital signs’ variability patterns | Relative extrema |
| **Oxygen saturation events (SaO_2_^g^)** | | | | |
|  | **Desaturation events^h^** | | | |
|  |  | 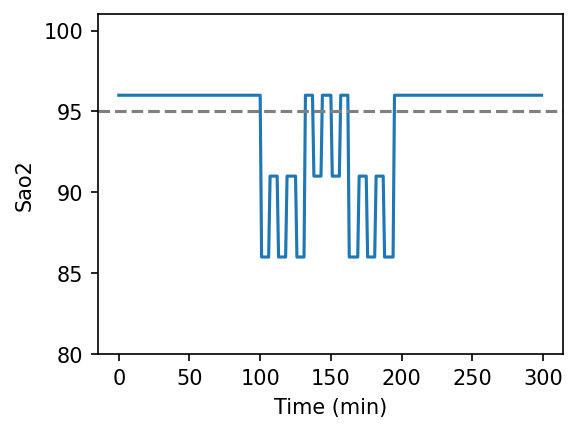 | 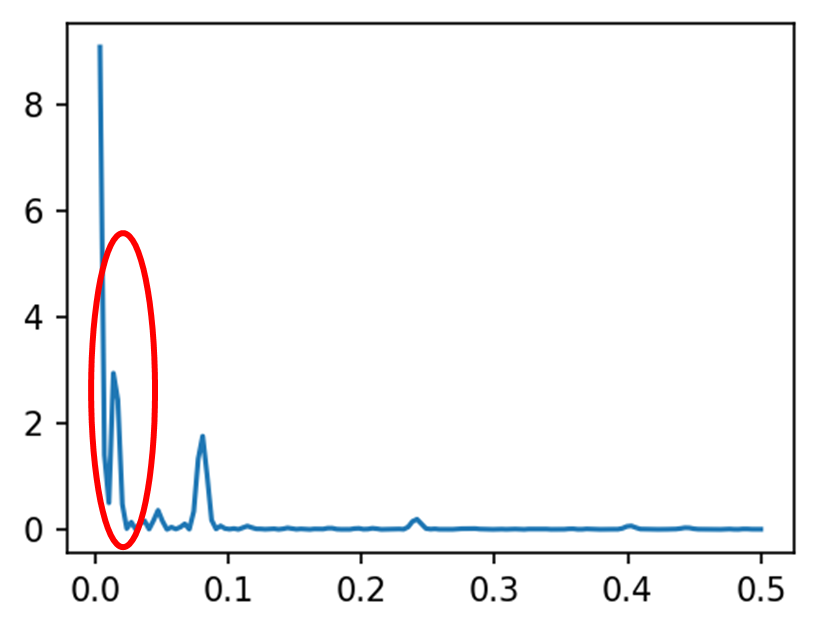   - Frequency domain: identify oxygen desaturation events using the positions of relative extrema | Relative extrema |
|  | **Saturation events^i^** | | | |
|  |  | 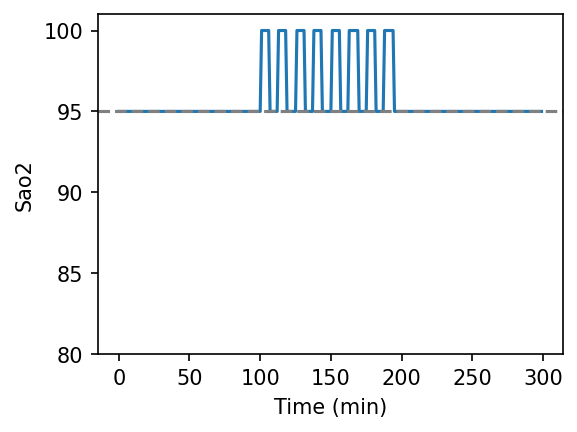 | 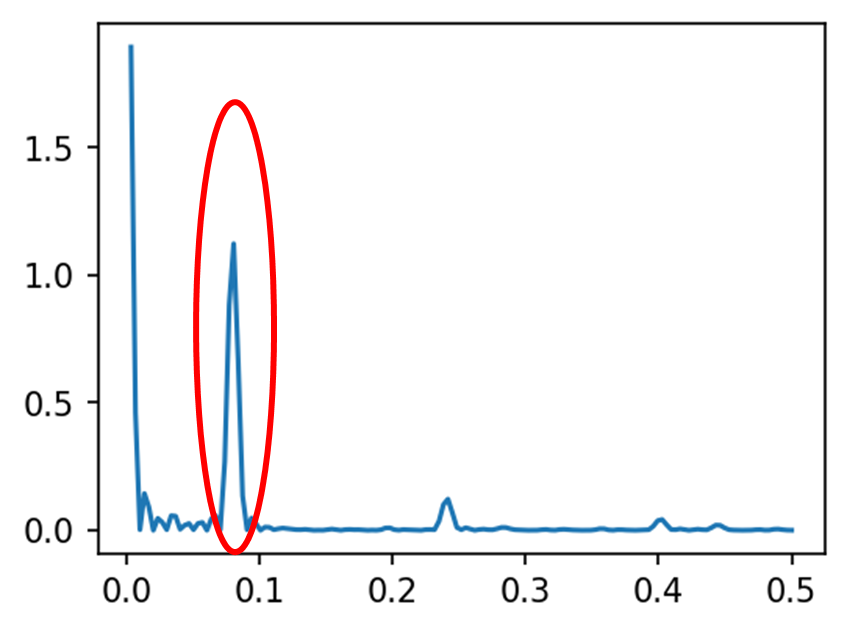   - Frequency domain: identify oxygen saturation events using the positions of relative extrema | Relative extrema |

^a^Hong et al [9], Zimmerman et al [12], Subbe et al [33], Bhogal and Mani [34], Donnelly [35], Nguyen et al [36], Pittappilly et al [37], Rajendra Acharya et al [38], and Ubaid et al [39].

^b^The patterns depicted in the plots are simplified representations. The time-series data from intensive care unit patients’ digital health records consist of a complex blend of various patterns.

^c^ICU: intensive care unit.

^d^Overall stability: the fluctuations in vital signs are generally associated with adverse events and increased mortality rate.

^e^Sudden change: an indicator of unfavorable health conditions.

^f^Short-term variabilities are fast changes in vital signs and are associated with various adverse events (eg, preventricular contraction, sick sinus syndrome, and atrial fibrillation). Long-term variabilities are slower fluctuations in vital signs and are associated with various adverse events (eg, congenital heart block, left bundle branch block, and ischemic or dilated cardiomyopathy).

^g^SaO_2_: arterial oxygen saturation.

^h^Desaturation events constitute a drop in blood oxygen level and are associated with unfavorable health conditions.

^i^Saturation events constitute a rise in blood oxygen level and are associated with favorable health conditions.
