# Supplementary material for: Intensive Care Unit Patient Outcome Prediction Using ν-Support Vector Classification and Stochastic Signal Processing–Based Feature Extraction Techniques: Algorithm Development and Validation Study
Source: JMIR AI. 2025 Aug 26;4:e72671. doi: 10.2196/72671 (PMC12421204; doi:10.2196/72671)
Supplement: Multimedia Appendix 2 [file ai_v4i1e72671_app2.docx]

## Multimedia Appendix 2

### A.1 Experiment Setup and Parameter Selection

The probabilistic classification problem $\hat{y}=arg max_{y} Pr(Y=y|X)$ described in Section 3.3 is implemented as follows: ν-SVC defines a hyperplane function, $f$, to make predictions, $f(x_{i})=1,$ if${f(x_{i})(w}^{T}\cdot x+b)\geq\rho-\xi_{i}; f(x_{i})=-1$otherwise. The ν-SVC does not explicitly predict probabilities. The probability of each class is estimated by cross-validation. The parameter $\theta=\{w, b\}$ can be obtained by optimizing $\min_{w, b}\frac{1}{2}w^{T}w-\nu p +\frac{1}{l}\sum_{i=1}^{l} \xi_{i}$.

**Relative extrema of frequency spectrums.** We conduct a prior experiment to select the parameters (i.e., *n* and *ε*) for relative extrema (Section 3.2) for the frequency spectrums. The AUC scores are shown in Table A1. When *n = 5*, the classifier achieves the best AUC most of the time. Thus, *n* is set to 5. With different ε, we can extract different features. Our proposed feature set includes different relative extrema features extracted using various ε (i.e., $\varepsilon\in\{2,4,8\}$).

Table A1. Parameter Selection for n and ε

| AUC | ε = 2 | ε = 4 | ε = 8 |
| --- | --- | --- | --- |
| n = 3 | 0.745 | 0.752 | 0.750 |
| n = 5 | 0.758 | 0.763 | 0.764 |
| n = 7 | 0.757 | 0.759 | 0.774 |

**Extreme values on vital signs.** As statistical features, we take the extreme values on the vital signs through the moving window method (Section 3.2). The window size is set to 10, which covers vital signs for 50 minutes. According to the medical professional’s recommendations, the selected window size is large enough to estimate the local mean and variance of the vital signs. The parameter $n$ is set to be 5 empirically because 43.43% of the vital signs have at least 5 extreme values in the moving windows. Such settings can include as many useful features as possible, while producing as few missing values as possible.

**Power-in-band.** Before we present the parameter setting of the power-in-band features, we first highlight the importance of power-in-band features from two aspects. First, power-in-band is the summary measure of the “strength” of a signal (i.e., vital signs are defined as signals in our research). (1) In signal processing, a signal is viewed as a function of time. “Power of a signal” is used to represent “strength of the signal”. (2) In signal detection techniques, the strength of a signal (also known as energy) is often considered as the computation of the area under the square of the signal $E_{signal}=\sum_{n=-\infty}^{\infty} {|signal(n)|}^{2}$ (see Figure A1). According to Parseval’s theorem (Figure A2), the energy of the time domain signal is equal to the energy of the frequency domain transform.

| 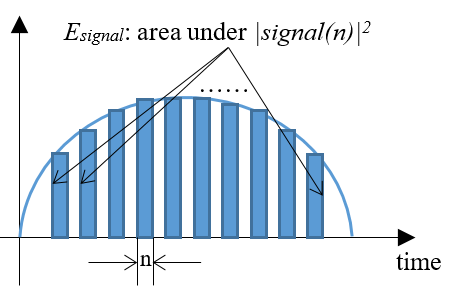 | 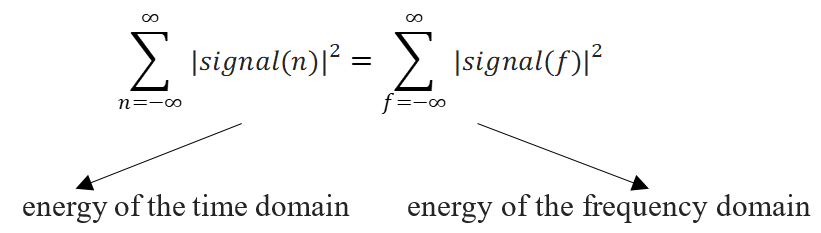 |
| --- | --- |
| Figure A1. Strength (Energy) of the Signal | Figure A2. Parseval’ Theorem |

“Power” is also the measure of signal strength, which is defined as the amount of “energy” consumed *per unit time* $P_{signal}=\lim_{N\to\infty}\frac{1}{2N+1}\sum_{n=-N}^{n=N} {|signal(n)|}^{2}$. Similarly, according to Parseval’s theorem, the power in a signal when expressed in the time domain is equal to the power of that same signal when expressed in the frequency domain. The power-in-band feature summarizes the contribution of the given frequency band (i.e., frequency range) to the overall power of the signal, which contains important patterns of patients’ vital signs. Meanwhile, power-in-band is a simple yet powerful dimension reduction method for ICU mortality prediction. (1) Power-in-band features are easy to extract and use. There are a variety of methods that can be used on the frequency spectrums once we convert patients’ time series of vital signs to the frequency domain. Power-in-band is one of the easiest to analyze. In practice, we compute the summation over different segments of a vector (i.e., the vector represents the frequency spectrums transformed from a vital sign). (2) Power-in-band features are useful for machine learning predictive analysis. The time/frequency representations (i.e. frequency spectrums generated by converting time series of vital signs to frequency domains) are of high dimensionality, making them unsuitable for use as classifier inputs. Power-in-band extracts the key characteristics from the time/frequency representations, resulting in a single number that describes a specific aspect of the time/frequency representations.

We conduct prior experiments to select the proper center frequencies $\omega_{c}$ and bandwidth $\omega_{bw}$ for the power-in-band features. We obtain fixed-length vectors of frequency spectrums after we transform the time-series of health digital traces to the frequency domain. We explore our model’s performance by splitting the vectors into $n$ bands of equal length ($n\in\{4，5，6\}$). The center frequencies $\omega_{c}$ and bandwidth $\omega_{bw}$ are determined by the number of bands $n$. For example, when the number of bands $n$ is set to $4$, there are four center frequencies; the center frequency is the middle point of each band and the width of each band $\omega_{bw}$ can be obtained accordingly. Similarly, when the number of bands $n$ is set to 5 or 6, we calculate the center frequencies and bandwidth correspondingly. The values of the parameters and the prediction results are summarized in Table A2. The experimental results show that when the number of bands $n$ is set to 5, the proposed method achieves the best empirical results. As a result, in this study, the center frequency $\omega_{c}$ is (1.67, 5.00, 8.33, 11.67, 15.00 ($*{10}^{-4}$)) and the bandwidth $\omega_{bw}$ is $1.67*{10}^{-4}$.

Table A2. Power-in-band Parameter Selection

| Number of bands | Bandwidth $\omega_{bw}$ | Center frequencies $\omega_{c}$($*{10}^{-4}$) | AUC |
| --- | --- | --- | --- |
| n = 4 | $2.08*{10}^{-4}$ | (2.08, 6.25, 10.41, 14.58) | 0.706 |
| n = 5 | $1.67*{10}^{-4}$ | (1.67, 5.00, 8.33, 11.67, 15.00) | 0.756 |
| n = 6 | $1.38*{10}^{-4}$ | (1.39, 4.17, 6.94, 9.72, 12.50, 15.26) | 0.707 |

**Parameters of Classifiers.**The parameters of the traditional machine learning classifiers used in this work are grid-searched for different patient cohorts. For CNNs, we followed the network architectures and parameters of [17], [18]. For LSTM [19] and GRU [42], we grid-searched parameters as shown in Table A3 since the authors did not provide implementation details. The parameters are shown in Table A3.

Table A3. Classifiers’ Parameters

| **Method** | **Parameters** |
| --- | --- |
| Decision Tree [22] | n_estimator: 400, 800, 1600; max_depth: 2, 4, 8, 16, 32 |
| Random Forest [23] | n_estimators: 400, 800, 1600; max_depth: 2, 4, 8, 16, 32; criterion: gini, entropy |
| Logistic regression [25] | Penalty: l1; C: 0.005, 0.01, 0.05, 0.1 |
| Gradient Boosting [24] | n_estimator: 400, 800, 1600; base_estimator: decision tree; max_depth: 2, 4, 8, 16, 32 |
| GRU [42] | The authors did not provide implementation details; therefore, parameters were grid searched.  Num_hidden_layers: 1, 2, 3; hidden_layer_size: 5, 10, 15; Optimizer: Adam (learning rate lr = 0.001). |
| CNN [18] | Follow the network architectures and parameters of S. Y. Kim et al. (2019). |
| CNN [17] | Follow the network architectures and parameters of Caicedo-Torres and Gutierrez (2019). |
| LSTM [19] | The authors did not provide implementation details; therefore, parameters were grid searched.  Num_hidden_layers: 1, 2, 3; hidden_layer_size: 5, 10, 15; Optimizer: Adam (learning rate lr = 0.001). |
| ν-SVC | Parameters were grid searched for different patient cohorts. ν:0.01, 0.1, 0.5; kernel: linear. |

**Time Series Models for ICU Outcome Prediction.** Time series models (e.g., ARMA and ARIMA) are employed to forecast the observation at time ($t+1$) based on historical data from the previous time ($t$, $t-1$, $t-2$, …) recorded for the same observation (i.e., the identical vital sign signal from a specific patient). These models are not suitable for direct application in binary classification tasks, such as ICU outcome prediction. To adapt the time series model for classification purposes, we follow previous research [25] and leverage the coefficients of time series models as inputs for a machine learning model. Nevertheless, the order of a time series model has to be determined by the statistical characteristics for a specific time series (e.g., one time series of vital signs for one patient) and a fixed order of time series model is required for all patients to ensure input features have the same dimension for the classification task, which limits the predictive power of the time series forecasting models in ICU outcome prediction.

Specifically, the “order of a time series model” delineates a set of parameters crucial for achieving optimal time series (e.g., vital sign signal) forecasting performance. Taking ARIMA($p$, $d$, $q$) as an example, the order of ARIMA includes: $p$, representing the number of lag observations; $d$, indicating the number of times the raw observations are differentiated; and $q$, denoting the size of the moving average window. In our study, ARIMA models were applied to vital signs, and the model parameters were initially determined for each patient’s individual vital sign by selecting values that minimized the AIC. Consequently, to achieve the lowest AIC, different values for $p$, $d$, and $q$ were assigned to each vital sign for every patient.

For example, an ARIMA($p=0$, $d=1$, $q=0$) model is $X_{t}=X_{t-1}+\varepsilon_{t}$, the coefficient of this model is {1}; an ARIMA($p=0$, $d=2$, $q=2$) model is $X_{t}={2X}_{t-1}-X_{t-2}+(\alpha+\beta-2)\varepsilon_{t-1}+(1-\alpha)\varepsilon_{t-1}+\varepsilon_{t}$, the coefficient of this model is {2, -1}. In our specific context, we utilize estimated ARIMA coefficients as inputs for machine learning classifiers to predict ICU outcomes (i.e., a binary classification problem). Given that each patient’s vital signs may possess distinct $p$, $d$, and $q$ values, there will be varying numbers of estimated coefficients for each patient. However, the majority of machine learning models require input features to possess uniform dimensionality across all data records. Consequently, to facilitate the binary classification prediction task, we establish a consistent order (uniform $p$, $d$, and $q$) for the time series model across all patients. This standardization ensures that input features for subsequent machine learning models maintain consistent dimensionality. To achieve this, we select the most frequently occurring $p$, $d$, and $q$ values among all patients for each vital sign.
