# Supplementary material for: Intensive Care Unit Patient Outcome Prediction Using ν-Support Vector Classification and Stochastic Signal Processing–Based Feature Extraction Techniques: Algorithm Development and Validation Study
Source: JMIR AI. 2025 Aug 26;4:e72671. doi: 10.2196/72671 (PMC12421204; doi:10.2196/72671)
Supplement: Multimedia Appendix 3 [file ai_v4i1e72671_app3.docx]

**Multimedia Appendix 3.** Statistical features and relations to health digital traces.

| Category and statistical measures | | | Motivation and relation to health digital traces |
| --- | --- | --- | --- |
| **Dispersion of vital signs** | | | While they are related, they provide different perspectives on the dispersion of the data. By including both the variance and SD, we gain a more comprehensive understanding of the variability of patients’ vital sign time series. The variance can help in identifying outliers or anomalies, whereas the SD helps understand the average spread or dispersion of the data. Together, they can provide a more robust picture of the variability within the dataset, which can be particularly useful in the context of summarizing complex health digital traces from ICU^a^ patients. |
|  | SD: $\sqrt{Var(V)}$ | The average of the squared differences from the mean. It provides a measure of how spread out the data are from the mean and is an important indicator of variability. |  |
|  | Variance: $Var(V)$ | The square root of the variance. It provides a measure of dispersion that is in the same units as the original data. This makes it more interpretable and relatable to the original values. |  |
| **The central tendency of vital signs** | | | Inclusion of the central tendency statistical measures provides a complete picture of a patient’s status over time. It captures what is “average” or “typical,” as well as how much variability there is and how extreme the extremes are. |
|  | Mean (*V_t_*) | The mean provides an understanding of the central tendency of the data. For example, the mean heart rate over a certain period might show that a patient generally has a high, low, or normal heart rate, which could be indicative of their overall health status. |  |
|  | Median (*V_t_*) | The median is less sensitive to outliers than the mean and might provide a more accurate reflection of what is “typical” for a given patient. For instance, if a patient’s heart rate spiked only a few times but generally remained stable, the mean heart rate might be skewed higher due to these few instances, whereas the median would remain stable. |  |
|  | Quantiles (*V_t_*) | They capture the distribution of values in a more granular way. For example, the 90th percentile of blood pressure readings can indicate how high blood pressure gets in the most extreme 10% of readings, which could be important for understanding the risk of certain complications. The IQR, which represents the middle 50% of the data, can also be useful in understanding the variability of the readings. |  |
| **The extreme values of vital signs** | | | The minimum and maximum values of patients’ vital signs can provide critical information about the extremities of a patient’s condition. In an ICU setting, this information can be vital in understanding a patient’s overall health trajectory, helping identify trends and potential health risks that may not be immediately apparent. |
|  | Minimum (*V_t_*) | The minimum values of a patient’s vital signs can indicate periods of significant health decline or instability. For example, a particularly low heart rate or blood pressure might indicate a medical emergency, such as cardiac arrest or septic shock. In predictive models, such minimum values could potentially flag a patient as being at high risk. |  |
|  | Maximum (*V_t_*) | The maximum values of a patient’s vital signs can serve a similar but opposite function. High heart rate, blood pressure, or fever might indicate severe stress, infection, or other significant health issues. In predictive models, high maximum values can help identify patients who are critically ill or at risk of becoming so. |  |
| **The first and last values of a vital sign** | | | The first and last values of patients’ vital sign time series can provide crucial insights into a patient’s health trajectory. |
|  | First: *V*_1_ | Vital signs upon arrival at the ICU, which reflect the patient’s condition upon admission to the ICU. They can provide a baseline to which subsequent measures can be compared. This could be particularly important if the patient’s condition changes rapidly after admission or if they were admitted due to a sudden, severe event such as a myocardial infarction or stroke. |  |
|  | Last: *V_N_* | Vital signs at the time of prediction, which can provide information about the patient’s most recent condition. This could be useful in assessing the effectiveness of interventions, monitoring progress, and predicting short-term outcomes. If the patient’s condition is deteriorating, the last values might be quite different from the earlier ones. |  |

^a^ICU: intensive care unit.
